# *Polygonatum sibiricum* polysaccharide-based hydrogels regulate macrophage mitochondrial homeostasis for the treatment of radiodermatitis

**DOI:** 10.1016/j.mtbio.2026.103520

**Published:** 2026-08-04

**Authors:** Wenfeng Li, Yawei Wang, Yang Xu, Ziyan Zhou, Yang Wang, Quanying Liu, Chunmeng Shi

**Affiliations:** a State Key Laboratory of Trauma and Chemical Poisoning, Third Military Medical University (Army Medical University), Chongqing, 400038, China; b School of Public Health, The Key Laboratory of Environmental Pollution Monitoring and Disease Control, Ministry of Education, Guizhou Medical University, Guiyang, 561113, China; c Department of Pulmonary and Critical Care Medicine, General Hospital of Western Theater Command, Chengdu, 610083, China

**Keywords:** Radiation-induced skin injury, Mitochondrial homeostasis, *Polygonatum sibiricum* polysaccharide, Hydrogel, Inflammation

## Abstract

Radiation-induced skin injury (RISI) is the major complication of tumor radiotherapy, and the key to its refractory nature lies in the persistent oxidative stress caused by ionizing radiation. It is noteworthy that this process is closely associated with the disruption of mitochondrial homeostasis in macrophages. Injured mitochondria not only amplify reactive oxygen species (ROS) production but also drive the polarization of macrophages toward a pro-inflammatory phenotype, thereby impeding tissue repair. However, effective strategies to restore macrophage mitochondrial homeostasis for RISI treatment remain limited. This study designed an immunomodulatory dual-network hydrogel (PCA) composed of oxidized *polygonatum sibiricum* polysaccharide, quaternized chitosan and sodium alginate. The PCA hydrogel exhibited excellent radioprotective effects on keratinocytes against ionizing radiation injury *in vitro*, promoted wound healing, facilitated macrophage M2 polarization, and suppressed inflammation in a mouse radiodermatitis model. It was further demonstrated that the radioprotective mechanism of PCA hydrogel in macrophages involves scavenging mitochondrial ROS, maintaining the integrity of mitochondrial ultrastructure and membrane potential, restoring oxidative phosphorylation to remodel energy metabolic homeostasis, and ameliorating ionizing radiation-induced structural and functional abnormalities of mitochondria. Notably, PCA inhibited the cytoplasmic leakage of mitochondrial mtDNA, alleviated radiation-mediated excessive inflammation, and promoted the polarization of macrophages toward a reparative phenotype. Collectively, PCA hydrogel protects mitochondrial homeostasis and modulates inflammatory responses, providing a promising candidate strategy for the treatment of radiodermatitis.

## Graphical abstract

**Figure:**
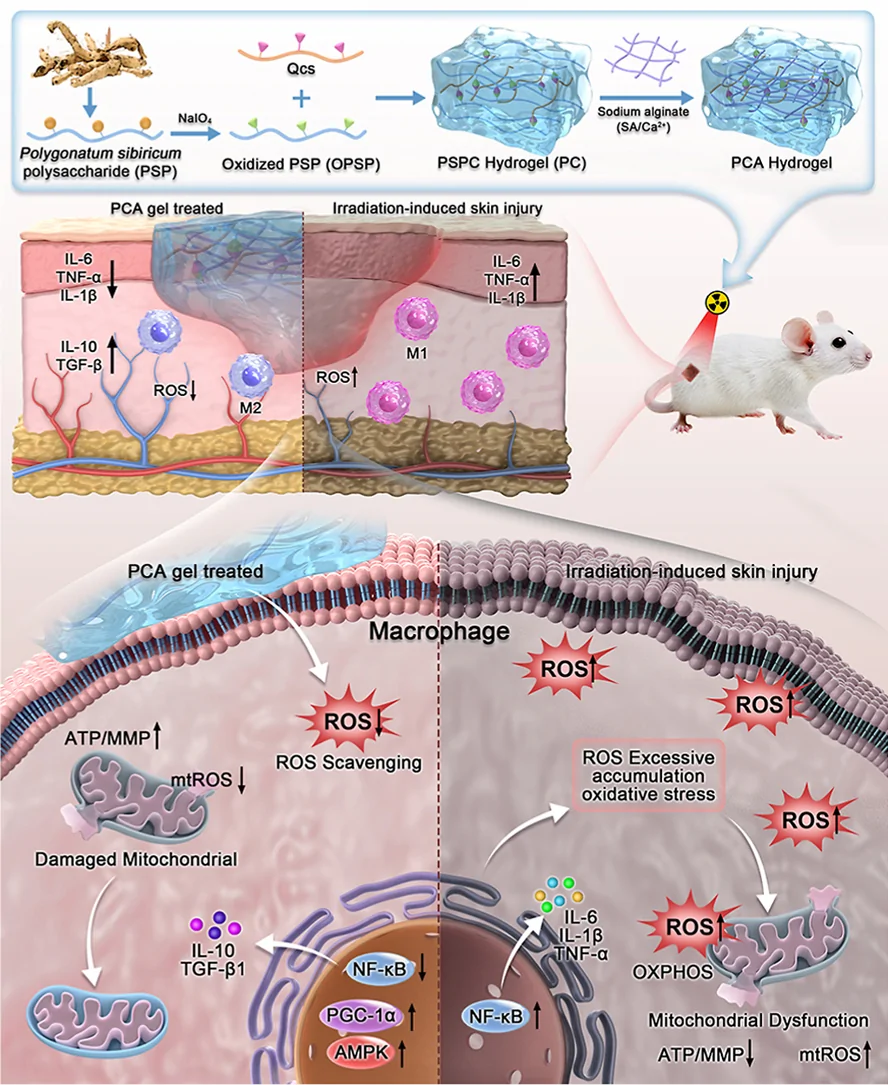

## 1 Introduction

Radiotherapy is among the most crucial cancer treatment modalities, and more than 50% of cancer patients require radiation therapy [1,2]. Although the efficacy of radiotherapy has been proven, the unnecessary radiation delivered to adjacent tissues remains an unavoidable issue in clinical practice [3]. As one of the most common side effects of radiotherapy, radiation-induced skin injury (RISI) has an incidence rate as high as 95% [4]. Early symptoms of RISI include mild erythema, dryness and desquamation of the skin, and pruritus. These injuries gradually worsen as the radiation dose increases, and wet desquamation, exudation, and even hemorrhagic scabs may occur. RISI can even lead to skin ulcers and necrosis in severe cases, imposing a significant economic burden on cancer patients and adversely affecting their clinical outcomes [5]. Therefore, exploring novel therapeutic targets and developing new treatment strategies for RISI are important.

During the pathogenesis of RISI, macrophage dysfunction and mitochondrial homeostasis imbalance are key factors impeding tissue repair [6,7]. Ionizing radiation triggers a surge in mitochondrial reactive oxygen species (mtROS), which impairs mitochondrial integrity and reduces oxidative phosphorylation and adenosine triphosphate production in macrophages [8,9]. Given that mitochondria are the central organelle for cellular energy metabolism, their dysfunction further compromises the oxidative phosphorylation capacity of macrophages, reduces adenosine triphosphate (ATP) production, and consequently disrupts the energy supply required for immune responses and tissue repair [5]. Furthermore, disordered mitochondrial homeostasis polarizes macrophages toward a proinflammatory phenotype, exacerbating local inflammation and suppressing their transition toward a reparative phenotype, ultimately delaying skin re-epithelialization and tissue regeneration [10]. While antioxidant therapies offer partial relief, a precise strategy that simultaneously clears mtROS, restores mitochondrial homeostasis, and regulates macrophage inflammatory phenotype remains elusive [11,12].

Currently, various mitochondria-regulated therapeutic strategies have been increasingly recognized for the treatment of RISI, including natural product-based, stem cell-based, and biomaterial-based interventions [5,[13], [14], [15]]. For instance, Rong et al. identified mitochondria as a key effector of radiation-induced tissue injury, with mitochondrial dysfunction mediating damage across multiple tissues through diverse mechanisms [16]. Recently, mitochondria-enriched nanovesicles derived from human umbilical cord mesenchymal stem cells have been shown to restore mitochondrial function and genomic stability in irradiated cells, offering a cell-free, mitochondria-targeted strategy for radiation-induced skin injury [14]. Meanwhile, the encapsulation of natural antioxidants such as astaxanthin into microalgae-derived extracellular vesicles, combined with hydrogel-based delivery systems, has also demonstrated potential in alleviating oxidative stress and preserving mitochondrial homeostasis [13]. These emerging strategies highlight the therapeutic promise of mitochondrial regulation in RISI management.

The traditional Chinese medicine *polygonatum sibiricum* polysaccharide (PSP) is a representative active component derived from *polygonatum cyrtonema* [17] that exhibits different biological activities, including immunomodulatory [18], antioxidant [19], and mitochondrial function-regulating properties [20]. Moreover, the ability of PSP to accelerate cutaneous wound healing via anti-inflammatory, antioxidant, and metabolic disorder-improving pathways was previously confirmed [21], suggesting its therapeutic potential for RISI associated with excessive oxidative stress and energy metabolism dysregulation.

Hydrogel dressings with excellent biocompatibility can maintain a moist wound microenvironment, serving as ideal materials for wound repair [22]. Using hydrogels as carriers to regulate macrophage polarization, improve mitochondrial function and relieve oxidative stress has been proven effective for wound healing [23,24]. Quaternized chitosan (Qcs) can form gel networks via Schiff base reaction between amino and aldehyde groups [25], and possesses good biocompatibility and antibacterial activity. Sodium alginate (SA) exhibits favorable biocompatibility, degradability, and water retention [26]. It can stabilize gel systems through ionic crosslinking and hydrogen bonding, and be used to construct dual-network hydrogels with tunable mechanical and degradation properties, showing great potential for the treatment of radiation-induced skin injury.

**Fig. 1:**
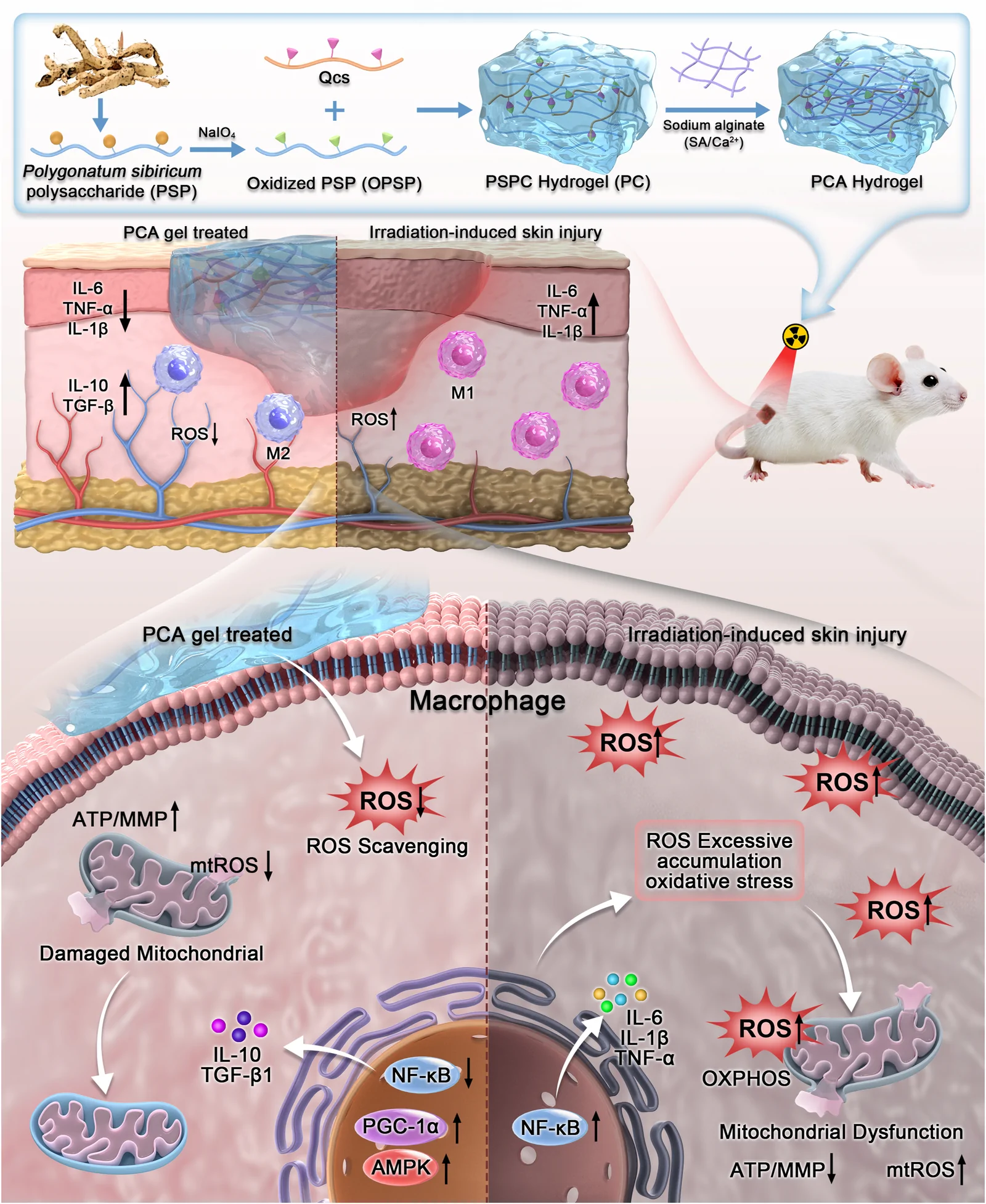
Schematic of the pathological change of irradiation-induced injury and the protective effect of PCA hydrogel.

This study innovatively modified PSP to introduce aldehyde groups, which formed an in-situ gel system via Schiff base reaction with Qcs. On this basis, a sodium alginate SA-calcium ion crosslinking system was further introduced to fabricate a PCA dual-network hydrogel for the treatment of RISI (Fig. 1). This PCA hydrogel exhibited excellent biocompatibility, injectability and mechanical properties. *In vitro* studies showed that the hydrogel regulated the survival and apoptosis of epidermal cells exposed to ionizing radiation. *In vivo* experiments verified that it accelerated skin wound healing, promoted collagen deposition at the wound site, and inhibited local inflammatory responses by modulating macrophage polarization. Further studies revealed that the PCA hydrogel not only scavenged excessive ROS in macrophages and restored mitochondrial membrane potential and ultrastructural integrity but also improved mitochondrial metabolism. Meanwhile, it suppressed the release of mtDNA into the cytoplasm, thereby blocking the downstream inflammatory cascade. This work provided not only new insights into the regulation of mitochondrial function in macrophages but also a novel therapeutic strategy with clinical translation potential for radiodermatitis.

## 2 Materials and methods

### 2.1 Cells and animals

The HaCaT cell was purchased from BeNa Culture Collection. HaCaT cells were incubated in RPMI 1640 supplemented with 10% FBS and 1% penicillin and streptomycin. Primary peritoneal macrophages (PMs) were isolated from C57BL/6J mice and cultured in DMEM supplemented with 10% FBS. All the cells were cultured in a humidified incubator at 37 °C with 5% CO_2_.

Male Balb/c (20∼22 g, 8 weeks old) and C57BL/6J mice were provided by the Army Medical University Animal Center. All animal experimental procedures were authorized by the Laboratory Animal Welfare and Ethics Committee of Army Medical University (approval no. AMUWEC20230024). The experimental animals were euthanized by cervical dislocation.

### 2.2 Synthesis of oxidized *polygonatum sibiricum* polysaccharide (OPSP)

PSP is a natural bioactive polysaccharide with antioxidant and immunomodulatory properties. For the synthesis of oxidized *polygonatum sibiricum* polysaccharide (OPSP), the hydroxyl groups of *polygonatum sibiricum* polysaccharide (PSP) were partially oxidized to aldehyde groups with sodium periodate (NaIO_4_). In brief, 10 g of PSP was first dissolved in 500 mL of ddH_2_O with magnetic stirring followed by 30 min of sonication to ensure complete dissolution. Afterward, 0.6 g of NaIO_4_ was dissolved in 20 mL of ddH_2_O, and this solution was added dropwise to the PSP solution. The reaction mixture was stirred for 2 h at 4 °C in the dark. Next, 5 mL of ethylene glycol was added, and the reaction was continued for 30 min to terminate the oxidation reaction. Finally, the mixture was dialyzed against distilled water using a dialysis membrane (MWCO: 300 Da) for 3 days, and the water was changed twice daily. The reaction product was finally frozen at −20 °C and dried. The oxidation process introduces aldehyde groups into the polysaccharide backbone while preserving the bioactive structural features of PSP, enabling OPSP to function as both a bioactive component and a crosslinkable building block.

### 2.3 Preparation of the PC and PCA hydrogels

The PCA hydrogel was prepared via a two-step crosslinking process. For PC hydrogel preparation, OPSP was dissolved in 1 mL of ddH_2_O and sonicated to generate a homogeneous solution with OPSP mass concentrations of 1%, 1.5% and 2%. Subsequently, 75 mg of Qcs was added to the OPSP solution with vortexing at high speed to form the PC hydrogel. The Qcs (degree of substitution 95%, viscosity 10-100 mPa s at 20 °C) was obtained from Macklin Biochemical Co., Ltd. The weight-average molecular weight (Mw) of Qcs was determined by gel permeation chromatography (GPC) to be 1991 g/mol, and the detailed GPC data are provided in Fig. S1 and Table S1. For PCA hydrogel preparation, to enhance the mechanical properties and sustained-release performance of the PC hydrogel, a secondary alginate-Ca^2+^ network was introduced. Briefly, different volumes (0.1, 0.25 and 0.5 mL) of a 15 mg/mL Sodium alginate (SA) solution were added to 1 mL of the PC hydrogel and the components were completely mixed. Then, approximately 100 μL of 0.1 M calcium chloride solution was sprayed onto the gel surface to prepare the PCA gel.

### 2.4 Physicochemical characterization of the materials

The FTIR spectra of the materials and hydrogels were recorded using a Fourier transform infrared (FT-IR) spectrometer (Thermo Fisher Scientific, Nicolet iS50, USA). Freeze-dried chemical material powders were directly placed on the crystal window after analysis of air absorption as the background. The dried hydrogels were first cut into thin shapes and then placed on the collection window for analysis. All the samples were detected in the wavenumber range of 400 to 4000 cm^−1^, and 32 scans were acquired.

The UV-Vis spectra of PSP and OPSP were acquired using a spectrophotometer (UV-3600 Plus, Japan) in the wavelength range of 200 to 400 nm. In brief, the UV absorbance of water was first recorded as a blank control. Afterward, sample solutions of PSP and OPSP (0.5 mg/mL) were dissolved in ddH_2_O and vortexed to form homogeneous solutions for analysis.

The ^1^H nuclear magnetic resonance (^1^H NMR) spectra of PSP and OPSP were recorded using a Bruker Avance III 600 spectrometer (Bruker, Germany) for chemical structure analysis. Briefly, 6 mg of PSP or OPSP powder was dissolved in 0.5 mL of deuterated water (D_2_O, 99.9% deuterium purity). The solutions were vortexed for 5 min to complete dissolution and then transferred to a standard 5 mm NMR tube (Synthware, China). ^1^H NMR spectra were acquired with the following parameters: a resonance frequency of 600 MHz, a relaxation delay of 2 s, 16 scans, and a spectral width of 12 ppm at room temperature. The spectral data were processed using MestReNova software.

The monosaccharide composition of the polysaccharide was determined by 1-phenyl-3-methyl-5-pyrazolinone (PMP) derivatization, which is applicable for reduced monosaccharides [27,28]. Detailed experimental procedures are provided in the supplementary materials.

### 2.5 Physicochemical characterization of the hydrogels

After freeze-drying, the PC, PCA and SA hydrogels were cut into small pieces and sputter-coated with gold for 15 s. The surface morphology of the hydrogels was observed by scanning electron microscopy (SEM; ZEISS Crossbeam 340) at an accelerating voltage of 5 kV. The pore size distribution of the hydrogels was quantified using ImageJ, with at least 50 pores measured per sample, and the results were statistically analyzed with Origin software.

The rheological characteristics of the hydrogels were analyzed using a Haake MARS Rheometer (HAAKE MARS 40, Thermo). The hydrogels were loaded between parallel plates with a diameter of 10 mm and a gap of 1 mm. The gelation time was determined by measuring the elastic modulus (G′) and viscous modulus (G″) during a 60 s time sweep at an angular frequency of 1 Hz at 25 °C. To analyze the gel viscosity, G′ and G″ were measured across a strain range of 0-100% at 1 Hz and 25 °C for 60 s. The critical point of the hydrogels was determined via strain amplitude sweep, and the self-healing capacity of the hydrogel was measured under a continuous strain sweep with alternating large and small oscillation forces between 1% and 500% over 20 s at 25 °C. Furthermore, the adhesive capacity of the PCA hydrogel of different shapes to fresh porcine skin was assessed.

The *in vitro* swelling characteristics of the hydrogels were measured using a weight difference method. The fresh hydrogels were weighed (W_*0*_) and immersed in PBS (pH 7.4) at 37 °C. At different time points, the hydrogels were removed from the PBS, gently blotted with filter paper to remove surface moisture, and weighed (W_*t*_). This process was repeated until the gel weight stabilized. The swelling ratio was calculated as follows:

$$\text{Swelling}\;\text{rate}\;\left(\%\right)=\left(W_{t}-W_{0}\right)/W_{0}\times 100\%\text{.}$$

The porosity of the hydrogel was determined by the ethanol displacement method. First, the initial mass (M_*1*_) of the hydrogel sample was determined after drying. Afterward, the sample was immersed in anhydrous ethanol (≥99.5%), and the total volume was recorded as V_*2*_. After the sample was degassed under vacuum for 2 h, it was immediately weighed to obtain the saturated mass (M_*2*_), and the absolute volume of ethanol was recorded as V_*1*_. The volume of ethanol absorbed by the hydrogel was calculated as V_*h*_ = (M_*2*_ - M_*1*_)/0.789, and the porosity (P) of the hydrogel was calculated as P = V_*h*_/(V_*2*_ - V_*1*_) **×** 100%. Each sample was measured in triplicate to determine the porosity.

To evaluate the *in vitro* release behavior of OPSP from PC and PCA hydrogels, release experiments were performed on both hydrogels. Briefly, OPSP was fluorescently labeled with FITC by slowly adding FITC solution dropwise to a 2% OPSP solution, followed by stirring at 4 °C for 4 h and dialysis against PBS at 4 °C for 24 h. The labeled product was collected and centrifuged before use. PC and PCA hydrogels were then prepared using the FITC-OPSP solution. 1 mL of each hydrogel was placed into a dialysis bag containing 8 mL of PBS, and immersed in an EP tube containing 30 mL of PBS, followed by shaking at 37 °C and 50 rpm for 72 h. At predetermined time points (10 min, 20 min, 40 min, 1 h, 2 h, 4 h, 6 h, 12 h, 24 h, and 48 h), 2 mL of the external dialysis solution was withdrawn for analysis, and an equal volume of fresh PBS was replenished. Separately, 1 mL of each hydrogel was completely dissolved in 8 mL of PBS to determine the total amount of FITC-OPSP encapsulated in the hydrogel. The fluorescence intensity was measured at an excitation wavelength of 480 nm and an emission wavelength of 520 nm using a Varioskan LUX multimode microplate reader (Thermo Fisher Scientific, USA). The concentration of FITC-OPSP was calculated based on a pre-established standard curve, and the cumulative release amount and release rate were subsequently calculated. All experiments were performed in triplicate.

To visualize the distribution of OPSP within the hydrogel network, FITC-labeled OPSP was used to prepare PC and PCA hydrogels, which were then uniformly spread onto glass coverslips. The green fluorescence was observed under a confocal laser scanning microscope, and 3D reconstruction was performed to analyze the spatial distribution of OPSP within the hydrogel matrix.

### 2.6 Cytotoxicity and hemocompatibility of the hydrogels

The toxicity of the hydrogels to HaCaT cells was determined using a Cell Counting Kit-8 (CCK-8, MCE) following the manufacturer's protocol. Briefly, 200 μL of each gel sample was prepared and individually immersed in 30 mL serum-free RPMI 1640 medium (Gibco, USA) at 4 °C for 12 h to obtain the hydrogel extracts. Cells were subsequently seeded into 96-well plates at a density of 5 × 10^3^ cells per well and cultured overnight. After the medium was removed, 100 μL of filtered hydrogel extract was added to each well for 1, 2, or 3 days of incubation. At each time point, 10 μL of CCK-8 working solution was added to the well and incubated for an additional 2 h. The OD_450 nm_ was measured using a microplate reader (Varioskan LUX, Thermo, USA). Cell viability was calculated as follows:

$$\text{Cell}\;\text{viability}\;\left(\%\right)=\left[\left({\text{OD}}_{sample}-{\text{OD}}_{blank}\right)\;/\;\left({\text{OD}}_{control}\;–\;{\text{OD}}_{blank}\right)\right]\times 100\%\text{,}$$

where OD_*sample*_, OD_*control*_, and OD_*blank*_ are the absorbance values of the gel extract, complete medium, and pure medium, respectively.

Furthermore, the cell survival rate after culture with the hydrogel extracts was evaluated using a Live/Dead viability assay kit (Beyotime, China). Cells were first seeded in 24-well plates and then incubated separately with 500 μL of various filtered hydrogel extracts. After 1, 2, or 3 days of incubation, the medium was removed and replaced with serum-free RPMI1640 medium containing 2 μM calcein-AM and 4 μM PI. The culture plates were incubated at 37 °C for 30 min. After staining, the wells were washed with PBS and then observed using a fluorescence microscope (Olympus, Japan) to analyze the live/dead status of the cells.

For cytoskeleton evaluation, HaCaT cells were cultured in 24-well plates and incubated with hydrogel extracts for an additional 24 h. After fixation with 4% paraformaldehyde (PFA) for 15 min, the cells were washed with PBS and permeabilized with 0.3% Triton X-100 for 10 min, followed by blocking with 2% (w/v) BSA for 30 min. The cells were then incubated with phalloidin (Actin-Tracker Red-555; Beyotime) at 4 °C overnight. After being washed with PBS, the nuclei were stained with DAPI (Beyotime, China), and the samples were visualized by confocal laser scanning microscopy (CLSM; Olympus).

The hemolytic effect of the hydrogels was evaluated following a previously described protocol with some modifications [29]. In brief, blood collected from the orbital sinus of a mouse was centrifuged at 3000 rpm for 15 min to isolate erythrocytes. The cells were then washed with fresh PBS until the supernatant became clear. The cells were subsequently incubated with saline ( negative control), ddH_2_O ( positive control) or different volumes of the PCA hydrogel extract at 37 °C for 2 h. In addition, OPSP solutions at various concentrations (1, 2, 3, 4, 5, and 6 mg/mL) were tested using the same protocol. The samples were subsequently centrifuged at 3000 rpm for 10 min, after which the absorbance of the supernatant was measured at 540 nm. The hemolysis rate was calculated as follows:

$$\text{Hemolysis}\;\left(\%\right)=\left[\left({\text{OD}}_{t}-{\text{OD}}_{b}\right)\;/\;\left({\text{OD}}_{p}-{\text{OD}}_{b}\right)\right]\times 100\%\text{,}$$

where OD_*t*_, OD_*b*_, and OD_*p*_ are the absorbance values of the hydrogel extract, negative control, and positive control, respectively.

### 2.7 Antioxidant capacity of the materials

The *in vitro* antioxidant capacity of PSP was evaluated using a 2,2-diphenyl-1-picrylhydrazyl (DPPH) free radical assay and a 2,2′-azino-bis (3-ethylbenzothiazoline-6-sulfonic acid) (ABTS) radical cation scavenging assay. Briefly, a 0.1 mM DPPH (Acmec, China) solution was prepared in ethanol, and PSP solutions of different concentrations (1, 2, 3, 4, 5 and 6 mg/mL) were prepared in water. Subsequently, 50 μL of each filtered PSP solution (with water serving as the blank control) was added to 950 μL of DPPH working solution for incubation in the dark at room temperature overnight. After incubation, the absorbance was measured at 517 nm using a UV-Vis spectrophotometer. The DPPH radical scavenging rate was calculated as follows:

$$\text{Scavenging}\;\text{rate}\;\left(\%\right)=\left[\left(A_{0}-A_{1}\right)/A_{0}\right]\times 100\%\text{,}$$

where A_*0*_ is the absorbance of the blank group and A_*1*_ is the absorbance of the PSP sample group.

The ABTS assay (Macklin, China) followed a similar protocol to that of the DPPH assay, with some modifications. The reaction mixture was incubated at room temperature for 1 h, after which the absorbance was measured at 734 nm. The ABTS radical scavenging rate was calculated using the same formula as that for the DPPH scavenging capacity.

### 2.8 Evaluation of the *in vitro* radioprotective effects of the hydrogels

#### 2.8.1 Cell proliferation and migration

Cell proliferation was assessed using a cell colony formation assay. Briefly, HaCaT cells were seeded into 6-well plates at a density of 400 cells per well and cultured overnight. The cells were then treated with fresh medium containing SA, PC, or PCA hydrogel extracts or with PBS (as a control) for 24 h. The cells were subsequently exposed to 4 Gy of X-ray irradiation (KUBTEC, ELL160 X-ray). After further incubation for 5 days, the cells were fixed with 4% PFA and stained with crystal violet (Beyotime, China). Cell colony images were captured, and the cell survival rate was calculated as follows:

$$\text{Survival}\;\text{rate}\;\left(\%\right)=W/W_{0}\times 100\%\text{,}$$

where W and W_*0*_ represent the numbers of cell clones in the experimental group and control group, respectively.

Cell migration was assessed using an *in vitro* wound healing assay. Briefly, HaCaT cells were seeded in 6-well plates at a density of 3 × 10^5^ cells per well and treated with various hydrogel extracts for 24 h to form a confluent monolayer. After irradiation with 30 Gy of X-ray, a “+” shape wound was created in the monolayer using a sterile 100 μL pipette tip, after which the samples were washed with PBS to remove dislodged cells. The cells were subsequently further cultured, and images were captured at 0 h, 18 h and 30 h. The wound area was calculated by ImageJ software, and the wound healing ratio was calculated as follows:

$$\text{Wound}\;\text{healing}\;\text{ratio}\;\left(\%\right)=\left(A_{0}-A_{t}\right)/A_{0}\times 100\%\text{,}$$

where A_*0*_ and A_*t*_ represent the wound area at the initial and indicated time point, respectively.

#### 2.8.2 Evaluation of the ability of the hydrogel to inhibit radiation-induced apoptosis

To evaluate apoptosis, cells were seeded into 24-well plates and incubated with various hydrogel extracts for 24 h. The cells were then exposed to 30 Gy of X-ray irradiation, followed by an additional 48 h of culture. After digestion with EDTA-free trypsin, the cells were harvested and washed twice with ice-cold PBS. Subsequently, 100 μL of binding buffer, 5 μL of Annexin V-FITC (YEASEN, China), and 10 μL of PI staining solution were added to the cell suspension with gentle mixing. After 15 min of incubation at room temperature in the dark, the samples were immediately analyzed by flow cytometry using the FITC and PI channels to determine the proportion of apoptotic cells.

To further evaluate the effect of this hydrogel against radiation-induced cell death, a live/dead staining assay (Beyotime, China) was conducted. The experimental protocol was the same as that described above, except that after incubation with the hydrogel extract for 24 h, the cells were then exposed to 30 Gy of X-ray irradiation. Live/Dead staining was performed 48 h post-irradiation, and the captured images were analyzed using ImageJ software.

#### 2.8.3 Evaluation of the ROS scavenging capacity of the hydrogels

For the ROS scavenging assay [12], HaCaT cells were seeded into 24-well plates at a density of 1 × 10^5^ cells per well and incubated overnight. The medium was then replaced with medium containing a hydrogel extract for an additional 24 h of incubation. The cells were subsequently exposed to 30 Gy of X-ray irradiation at a dose rate of 1 Gy/min. Eight hours after irradiation, the cells were treated with serum-free medium containing 1 μL/mL DCFH-DA (Solarbio, China) and incubated at 37 °C for 30 min. After being washed with fresh PBS, the ROS levels were visually analyzed using a confocal laser scanning microscope (Olympus, FV3000) and quantified using flow cytometry (BD Accuri C6) in the FITC channel, with 10,000 cells collected per sample for analysis.

### 2.9 Evaluation of the effects of the hydrogels on macrophages *in vitro*

To evaluate the effects of the hydrogels on macrophages *in vitro*, we first assessed the cytotoxicity of different concentrations of the hydrogel extracts via CCK-8 assays. PMs were isolated and seeded in 96-well plates at a density of approximately 85%, after which they were incubated with various hydrogel extracts for 24 h. Then, CCK-8 working solution was added, and the OD_450nm_ was measured. After the optimal concentrations of the gel extracts were determined, Live/Dead staining was carried out to evaluate hydrogel toxicity. Briefly, PMs were seeded in 24-well plates, incubated with the gel extracts in medium for different durations, and then stained for observation. The specific methods used were the same as those for the HaCaT cell experiments described above. To determine the appropriate irradiation dose and rate, cells were seeded in 96-well plates and exposed to 2, 4, 6, or 8 Gy at doses of 1 Gy/min and 2 Gy/min. CCK-8 assays were performed 24 h post-irradiation.

To assess phagocytic ability, PMs were seeded in 6-well plates and incubated with various hydrogel extracts for 24 h. Afterward, the samples were exposed to 4 Gy of X-ray irradiation at a dose rate of 1 Gy/min. After an additional 24 h of incubation, the medium was removed and replaced with 1 mL of fresh serum-free medium containing 1 μl of fluorescent microbeads for another 30 min of incubation. The cells were subsequently washed twice with PBS and imaged using a fluorescence microscope. The uptake rate was quantified using ImageJ software.

To evaluate the anti-inflammatory effect of the hydrogels on PMs, the cells were treated with various hydrogel extracts for 24 h, followed by exposure to 4 Gy of X-ray irradiation. After an additional 24 h of incubation, the cells were washed twice with cooled PBS and then lysed using TRIzol (Invitrogen). Total RNA was extracted and reverse-transcribed into cDNA using PrimerScript RT Mix (Thermo Scientific, USA). Gene expression was quantified using ChamQ SYBR Color qPCR Master Mix (Vazyme, China) on a Bio-Rad real-time PCR system using the primer sequences listed in Table S3. The gene expression levels were normalized to those of actin, and the fold change values were calculated via the 2^−ΔΔCt^ method. Furthermore, the cell culture supernatants were collected and subjected to enzyme-linked immunosorbent assays (ELISAs) according to the manufacturer's protocols to measure the secretion of inflammatory cytokines.

To assess the ability of the cells to scavenge intracellular ROS, PMs were first stained with DCFH-DA and then analyzed using fluorescence microscopy and flow cytometry. The experimental procedure was the same as that used in the above HaCaT cell experiments, but ROS levels were measured 12 h after the administration of 4 Gy of irradiation.

### 2.10 Mitochondrial function analysis on PMs

The mitochondrial membrane potential (ΔΨm) and mitochondrial reactive oxygen species (mtROS) levels were evaluated using a JC-1 assay kit and MitoSOX™ Red mitochondrial superoxide indicator (Beyotime, China), respectively. After being seeded into confocal dishes and treated with various hydrogel extracts, the cells were exposed to 4 Gy of irradiation and incubated for an additional 24 h. The cells were subsequently washed with PBS and incubated with JC-1 working solution (diluted 1:400 in serum-free medium) at 37 °C for 20 min. After the dye was removed and the cells were washed twice with PBS, the nuclei were stained with Hoechst 33342 (Beyotime, China) for 5 min. JC-1 monomers and aggregates were visualized on the basis of their characteristic green and red fluorescence, respectively. For mtROS detection, the cells were incubated with MitoSOX™ Red (diluted 1:3000) at 37 °C for 20 min. Fluorescence images were acquired using a confocal laser scanning microscope, and the fluorescence intensity was quantified using ImageJ software.

To further evaluate the whether mitochondrial homeostasis is causally required for the immunomodulatory effects of PCA, we performed a pharmacological inhibition experiment using rotenone (Beyotime), a specific mitochondrial complex I inhibitor. Briefly, macrophages were incubated with PCA for 24 h, followed by irradiation and immediate treatment with 1 μM rotenone. Mitochondrial membrane potential was assessed by JC-1 staining (Beyotime). Macrophage polarization was evaluated by immunofluorescence staining for CD86 (13395-1-AP), iNOS (ab178945), CD206 (ab64693), and ARG1 (93668s), and IL-10 levels in supernatants were measured by ELISA (CUSABIO, China). Detailed methods are provided in the Supplementary Information (Section 3, page S3).

The mitochondrial ultrastructure was observed by transmission electron microscopy (TEM). Briefly, after treatment with various gel extracts, PMs were harvested 24 h post-irradiation and fixed with 2.5% glutaraldehyde overnight at 4 °C. The cells were subsequently washed with PBS and fixed with 1% osmium tetroxide in the dark for 2 h at room temperature. After being washed twice with water, the samples were dehydrated through a graded ethanol series, infiltrated with acetone, and embedded in epoxy resin. Ultrathin sections (50-80 nm) were prepared using an ultramicrotome, after which the sections were stained with uranyl acetate and lead citrate. Finally, mitochondrial morphology was observed using TEM (FEI, USA).

Mitochondrial DNA (mtDNA) leakage into the cytosol was assessed by immunofluorescence staining. Following treatment, the PMs were washed with PBS and fixed with 4% paraformaldehyde for 15 min at room temperature. The cells were subsequently permeabilized with 0.3% Triton X-100 for 10 min and blocked with 5% BSA for 1 h at room temperature. The samples were then incubated with primary antibodies against DNA monoclonal antibody (AC-30-10) and TOM20 (ab186734) overnight at 4 °C. The cells were then washed three times with PBS before being incubated with the appropriate secondary antibody at room temperature for 2 h. After being washed with PBS, the nuclei were stained with DAPI. Images were acquired using a laser scanning confocal microscope (Olympus, FV3000) and quantified using ImageJ software.

To assess the leakage of mitochondrial DNA (mtDNA) into the cytoplasm, PMs were collected following different treatments. Cytoplasmic fractions were isolated by gentle lysis and differential centrifugation. DNA was then extracted from these fractions and subjected to qPCR with primers targeting the mitochondrial genes mt-Co3 and mt-Nd1. Cell metabolic status was evaluated by assessing intracellular ATP levels, the NAD^+^/NADH ratio and mitochondrial respiration. Following different treatments, ATP and NAD^+^/NADH concentrations were measured using corresponding commercial assay kits (Beyotime, China) according to the manufacturer's protocols. In addition, cellular mitochondrial respiration was measured using a Seahorse XFe96 analyzer (Agilent Technologies, USA). Briefly, PMs were seeded into XF96-well plates, treated with various hydrogel extracts for 24 h, and then exposed to 4 Gy of X-ray irradiation. After an additional 24 h of incubation with gel extract-containing medium, the medium was replaced with unbuffered Seahorse XF assay medium (pH 7.4) containing 1 mM pyruvate, 2 mM glutamine, and 10 mM glucose. The cells were then incubated at 37 °C in a CO_2_ free incubator overnight. Mitochondrial function was evaluated by sequential injection of modulators from the XF Cell Mito Stress Test Kit, which included oligomycin (1.5 μM), FCCP (1 μM), and a combination of rotenone and antimycin A (0.5 μM). The main parameters of mitochondrial function, including basal respiration, ATP production, maximal respiration, and spare respiratory capacity, were derived from the oxygen consumption rate (OCR) measurements and normalized to total protein content as determined by a BCA assay (Beyotime, China).

### 2.11 *In vivo* experiments

#### 2.11.1 Animal model establishment and treatment

To evaluate the therapeutic efficacy of the hydrogels, an RISI model was established using male Balb/c mice (7-8 weeks, 20-22 g). After isoflurane inhalation anesthesia, the dorsal hair was shaved, and a 2 cm × 2 cm area was outlined on the back. Subsequently, the mice were immobilized on a foam board and exposed to 40 Gy of X-ray irradiation at a dose rate of 2 Gy/min [30]. On day 7 post-irradiation, the mice were randomly assigned to five groups: control, SA, PC, PCA, and Kangfuxin (KFX; positive control) [31]. KFX, a clinically approved therapeutic agent for radiodermatitis in China, was selected as the positive control to provide a clinically relevant benchmark for evaluating the therapeutic performance of our PCA hydrogel. The treatments were administered topically to the wound area every other day for 14 days, and the wound areas were observed and photographed at specific time points. The wound closure rates of the different groups were quantified using ImageJ software and normalized to the wound size on day 7 post-irradiation. On days 7 and 14 after the initiation of treatment, skin tissue samples from the irradiated regions were harvested and fixed or frozen for subsequent analyses.

#### 2.11.2 Histopathological and immunofluorescence staining evaluations

The irradiated skin tissue samples were fixed in 4% PFA for 24 h, dehydrated and embedded in paraffin. Paraffin sections (2.5 μm) were subsequently prepared and stained with hematoxylin and eosin (H&E) (ZSGB-BIO, China) for histological assessment, Masson's trichrome (Solarbio, China) for collagen deposition, and Picrosirius red (Servicebio, China) for collagen typing analysis. Stained sections were imaged using a slide scanner (Olympus VS200) and quantified with ImageJ software.

For immunofluorescence staining, the paraffin sections were deparaffinized, rehydrated, and subjected to heat-mediated antigen retrieval in sodium citrate buffer at 95 °C. After cooling to room temperature, the sections were blocked with normal goat serum (ZSGB-Bio, China) at room temperature for 30 min and incubated overnight at 4 °C with specific primary antibodies. The sections were then washed with PBS, incubated with fluorescently labeled secondary antibodies for 2 h at room temperature, and stained with DAPI to visualize the nuclei. Primary antibodies against the following factors were used: TNF-α (ab6671), VEGF (AF5131), CD86 (13395-1-AP), and CD206 (AF2535).

#### 2.11.3 Quantification of inflammatory cytokine expression

For inflammatory cytokine analysis, skin wound tissue samples were collected on day 14, immediately immersed in nitrogen and then stored at −80 °C until analysis. The samples were subsequently lysed with RIPA lysis buffer (Beyotime, China) and homogenized at 4 °C. Then, the samples were centrifuged, and the protein concentration in the supernatant was determined with a BCA protein assay kit (Beyotime, China). The concentrations of inflammatory cytokines, including IL-1β, TNF-α, IL-6, IL-10 and TGF-β1 (CUSABIO, China), were measured using specific ELISA kits according to the manufacturer's protocols. Furthermore, total RNA from the samples was extracted from frozen wound tissues using TRIzol reagent, followed by reverse transcription into cDNA. The mRNA expression levels of inflammatory factors were quantified by RT-qPCR under consistent reaction conditions as previously described for the cellular experiments.

### 2.12 Statistical analysis

All the data are presented as the mean ± standard deviation (SD). Statistical analyses were performed by one-way ANOVA with a post hoc test using GraphPad Prism 8 (USA), with statistical significance defined as * p < 0.05, **p < 0.01, ***p < 0.001, and ****p < 0.0001.

## 3 Results

### 3.1 Synthesis and characterization of oxidized PSP (OPSP)

**Fig. 2:**
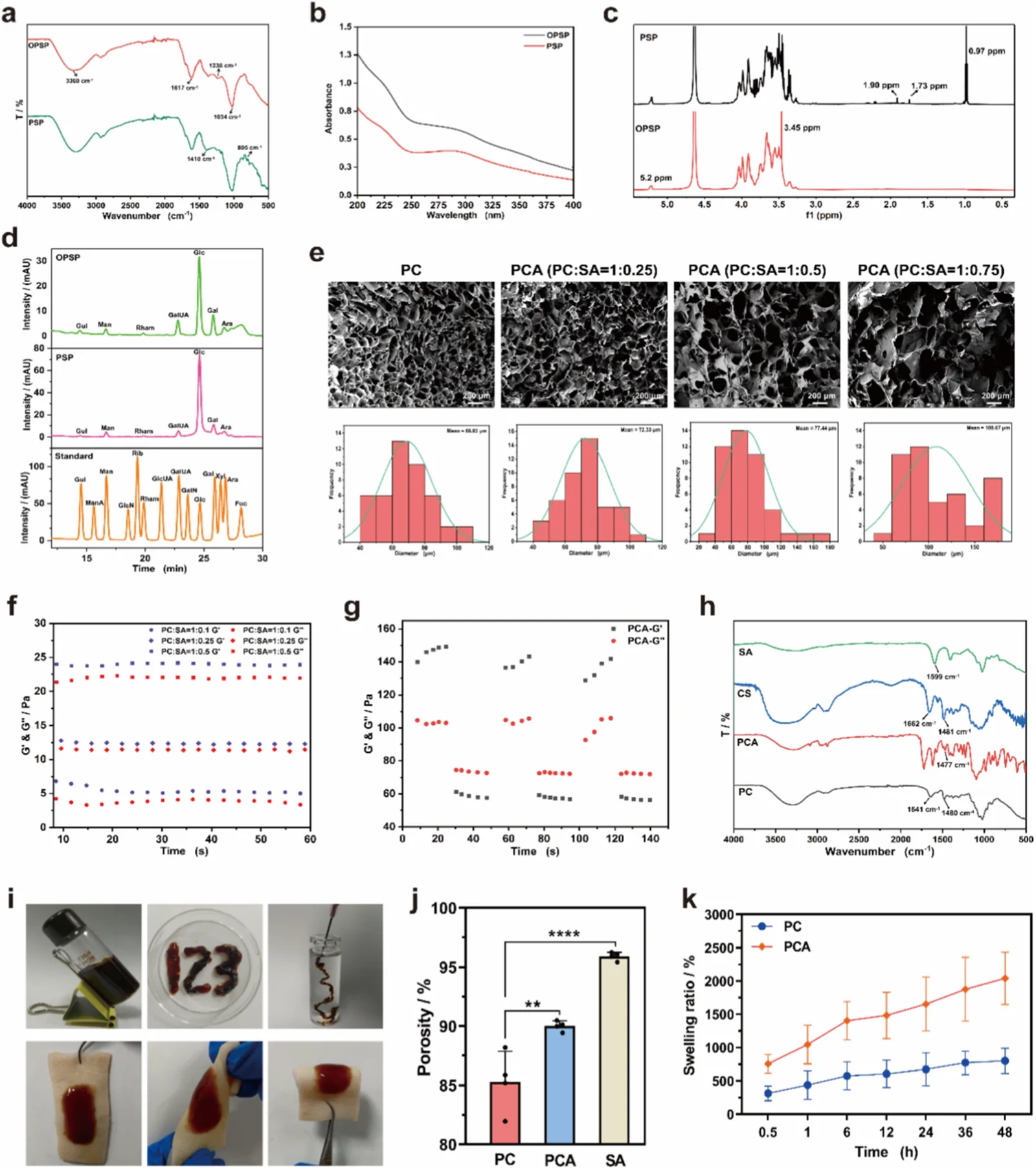
Characterization of OPSP and PCA hydrogel. (a-c) FT-IR spectra, UV-vis spectra, and ^1^H NMR spectra of PSP and OPSP. (d) HPLC of mixed monosaccharide standards and polysaccharides of PSP and OPSP. (e) SEM images and pore-size distributions of PC (2% w/v) and PCA hydrogels at PC-to-SA volume ratios of 1:0.25, 1:0.5, and 1:0.75. Scale bar, 200 μm. (f) Time-sweep rheology of PC and SA hydrogels at different volume ratios, showing G′ and G″ versus time over 60 s. (g) Strain-dependent rheology of PCA hydrogel (PC:SA = 1:0.25) under alternating shear strain (100% and 500%). (h) FT-IR spectra of PC, PCA, SA, and Qcs. (i) Photograph, injectability demonstration, and adhesion of PCA hydrogel to porcine skin. (j) Porosity measurement of PC and PCA hydrogels in ethanol solution. Data are presented as mean ± SD (n = 3). Statistical significance was determined using one-way ANOVA followed by Tukey's post-hoc test. **p < 0.01, ****p < 0.0001. (k) Swelling behaviors of PC and PCA hydrogels in PBS buffer over 48 h (n = 3).

To address the need for RISI treatment, we developed a PSP-based hydrogel. After oxidation with sodium periodate, OPSP possessed aldehyde groups on the side chains of the polysaccharide. The FT-IR spectra (Fig. 2a) show the attenuation of the O-H stretching vibration at 3360 cm^−1^ and the disappearance of the C-O stretching vibration at 1192 cm^−1^, indicating the modification of hydroxyl groups. In addition, the absorption at 817 cm^−1^, attributed to the alcohol-hydroxyl C-O group, also disappeared in OPSP, which suggested bond cleavage during oxidation [32]. Furthermore, the characteristic O-H and C-H angular vibrations of glucuronic acid at 1617 cm^−1^ and 1410 cm^−1^ remained in the spectra of both PSP and OPSP. The characteristic peak at 2934 cm^−1^ was assigned to the C-H stretching vibration of the methyl and methylene groups of the sugar ring, and the peak at 1740 cm^−1^ was assigned to the C=O bond stretching vibration [33]. The UV-vis spectra (Fig. 2b) of PSP and OPSP displayed negligible absorbance in the region between 200 and 400 nm, which suggested that the native material and oxidized PSP contain minimal protein and nucleic acid impurities [34].

The chemical structures of PSP and OPSP were further examined by ^1^H NMR spectroscopy (Fig. 2c). A distinct signal at 0.97 ppm in the PSP spectrum was attributed to the methyl (-CH_3_) protons of rhamnose (Rha) residues, which disappeared after sodium periodate oxidation because of the cleavage of the vicinal diols in the 1→2- or 1→4-linked Rha units. The disappearance of the acetyl methyl signals at 1.73 ppm and 1.90 ppm in the OPSP sample was attributed to alkaline side reactions leading to the hydrolysis of the acetyl groups [35]. The notable increase in the intensity of the signal at 3.45 ppm in the OPSP spectrum originated from enriched methylene protons in the glyceraldehyde structure (HOCH_2_-CHO), which were generated upon the cleavage of vicinal trihydroxy groups and suggested the presence of 1 → 4/1 → 6 linked glycosidic units. Compared with those in the PSP spectrum, the intensities of the signals in the 3.35-3.83 ppm region were weakened or eliminated in the OPSP spectrum after periodate oxidation, which resulted from the cleavage of oxidation-sensitive sugar rings and the disruption of the proton chemical environments [36]. The anomeric proton signals at 5.2 ppm and the signals of the sugar ring protons in the 3.9-4.0 ppm region remained stable in OPSP, confirming that the polysaccharide backbone is primarily composed of 1 → 3 glycosidic linkages [37] and indicating that the β-glucan core backbone linked via 1 → 3 bonds remained intact after sodium periodate oxidation.

The monosaccharide compositions of PSP and OPSP were determined by PMP precolumn derivatization HPLC (Fig. 2d and Table S2). Briefly, the contents of all the monosaccharides in PSP and OPSP were calculated by comparing the peak retention times with those of standard monosaccharides [28]. The results confirmed that Glc was the predominant monosaccharide, with molar percentages of 82.96% and 70.17% in PSP and OPSP, respectively. In addition, changes in the proportions of Man (from 2.86 to 2.92), GalUA (from 2.92 to 7.64), Gal (from 4.73 to 10.29) and Ara (from 3.09 to 5.55) were also observed upon the transformation from PSP to OPSP. In general, the monosaccharide profile of OPSP remained largely consistent with that of PSP, indicating that no major changes in composition occurred after sodium periodate oxidation.

### 3.2 Preparation and characterization of the hydrogels

PC hydrogels were fabricated through a Schiff base reaction between the amino groups of quaternized chitosan and the aldehyde groups of OPSP. First, the effect of the concentration of OPSP (1%, 1.5%, and 2% w/v) was evaluated in the presence of the same mass of Qcs. SEM (Fig. S2) revealed that the gels prepared with the 2% OPSP solution exhibited a uniform pore structure and a smaller diameter distribution (69.27 μm). Therefore, we selected 2% OPSP for subsequent experiments. To determine an appropriate mass of Qcs, the rheological properties were evaluated. The results revealed that 75 mg of Qcs per 1 mL of 2% OPSP solution resulted in an ideal gelation time and appropriate viscosity and shear resistance (Fig. S3a and 3b).

To improve the performance of the PC hydrogel, we introduced a gel system composed of sodium alginate and calcium ions to fabricate the PCA hydrogel. SEM analysis of the PCA gel showed a uniform pore size and diameter distribution (72.33 μm) similar to those of the PC hydrogel when prepared at a PC to SA volume ratio of 1:0.25 (Fig. 2e). In addition, the PCA hydrogel exhibited appropriate gelation time (G’ > G″) (Fig. 2f) and self-healing capacity within the strain range of 100%-500% (Fig. 2g). Therefore, a PC-to-SA volume ratio of 1:0.25 was selected for subsequent experiments. The successful construction of the hydrogel networks was confirmed via FT-IR spectroscopy. The characteristic peaks of Qcs at 1662 cm^−1^ (C=O stretching) and 1481 cm^−1^ (C-H bending) were observed in the product (Fig. 2h). Moreover, a new peak at 1641 cm^−1^ in the spectrum of the PCA hydrogel was attributed to C=N stretching vibrations, confirming the occurrence of the Schiff base reaction. Furthermore, an additional peak at 1599 cm^−1^ was detected in the PCA hydrogel spectrum, which matched the asymmetric stretching vibration of the SA carboxylate group (-COO-), verifying its successful incorporation into the network. Collectively, these results demonstrate the successful formation of a multicomponent hydrogel network via Schiff base reactions and the effective integration of SA.

The PCA hydrogel was successfully prepared within 30 s and exhibited excellent injectability (Fig. S4). Confocal 3D reconstruction of FITC-labeled OPSP revealed a continuous and uniform distribution within both PC and PCA hydrogels (Fig. S5). It could be extruded to form various patterns, indicating its adaptability to irregular wound surfaces. Moreover, this PCA hydrogel exhibited excellent extensibility on fresh pork skin and maintained firm adhesion even under bending, twisting and compression (Fig. 2i). This powerful adhesion ensures close contact with the wound bed, which helps maintain a moist environment and prevents bacterial invasion.

In addition, the swelling properties and porosities of the hydrogels were evaluated. The swelling capacity of the PCA hydrogel was significantly greater than that of the PC hydrogel (Fig. 2k), which was attributed to its increased porosity after the incorporation of the highly hydrophilic alginate network (Fig. 2j). This structure enables the composite matrix to absorb and retain more water internally, which is beneficial for its subsequent application.

To further evaluate the release behavior of OPSP from the hydrogels, *in vitro* release assays were performed using FITC-labeled OPSP (FITC-OPSP). As shown in Fig. S6, both PC and PCA hydrogels exhibited sustained release profiles without obvious burst release. The cumulative release of FITC-OPSP from the PCA hydrogel reached 67.86% at 6 h and 99.09% at 48 h, while that from the PC hydrogel reached 73.32% and 107.32%, respectively. The slower release rate observed in the PCA hydrogel may be attributed to the denser network structure formed by the alginate-Ca^2+^ crosslinking, which increases the diffusion resistance of the encapsulated OPSP. These results indicate that the PCA hydrogel serves as an effective sustained-release platform for OPSP delivery.

### 3.3 Biocompatibility of the PCA hydrogel

**Fig. 3:**
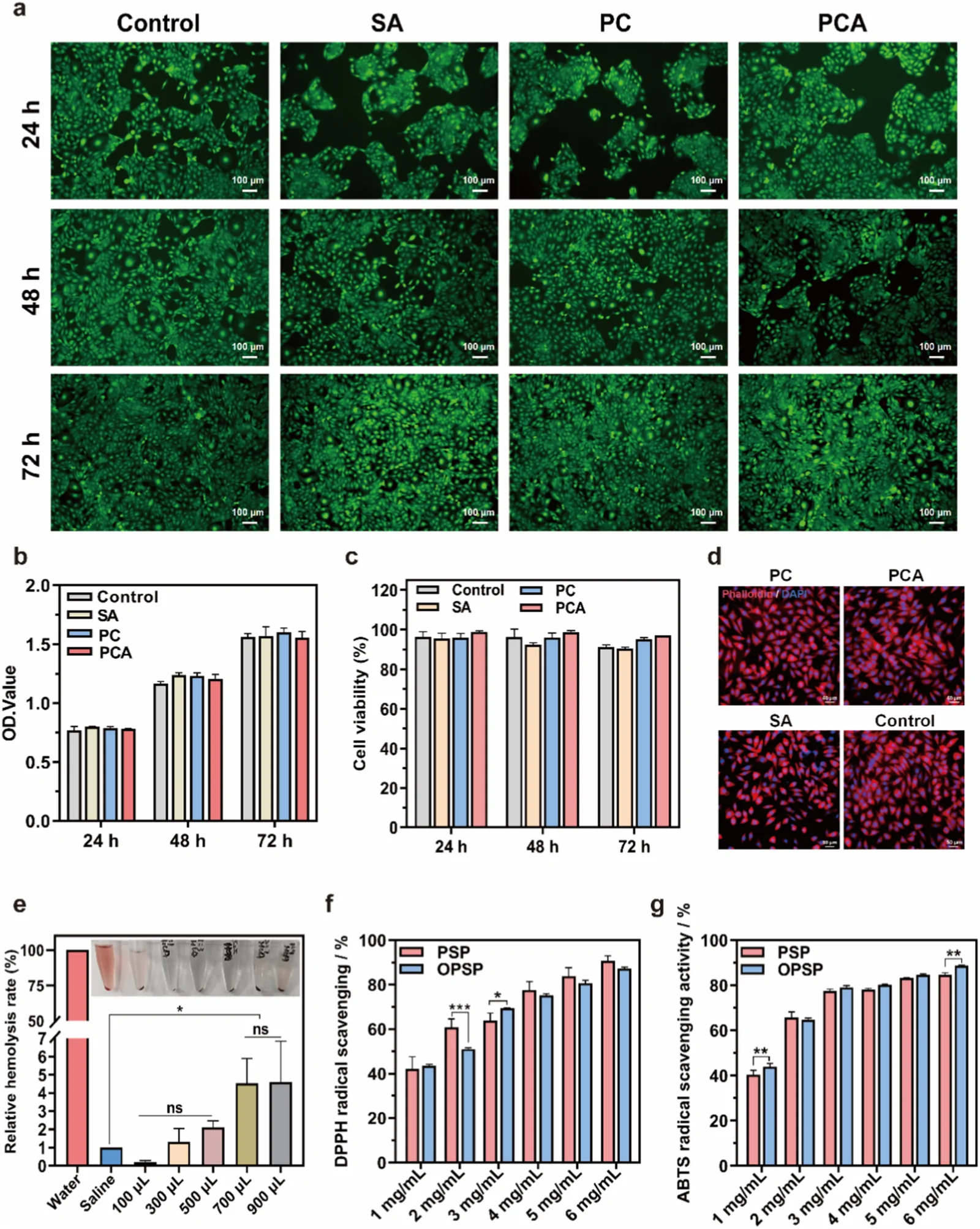
*In vitro* evaluation of hydrogel biocompatibility and antioxidant properties. (a) Live/Dead staining (Calcein-AM/PI) of HaCaT cells after incubation with hydrogel extracts for 24, 48, and 72 h, and (c) corresponding quantification. Scale bars, 100 μm. Images represent merged green (live) and red (dead) channel. (b) Viability of HaCaT cells incubated with hydrogel extracts for 24, 48, and 72 h. (d) Representative phalloidin staining of F-actin (green) after 24 h culture with hydrogels. Scale bar, 50 μm. (e) Hemolysis assay of PCA hydrogels at different ratios. Quantitative analysis of (f) DPPH and (g) ABTS radical scavenging activity after incubation with PSP and OPSP *in vitro*. Data are presented as mean ± SD (n = 3). Statistical significance was determined by one-way ANOVA followed by Tukey's post-hoc test. *p < 0.05, **p < 0.01, ****p < 0.0001. (For interpretation of the references to colour in this figure legend, the reader is referred to the Web version of this article.)

Excellent biocompatibility is critical for the clinical application of hydrogel-based wound repair dressings. To evaluate the biological safety of the hydrogels, we first investigated the viability of cells incubated with different concentrations of the hydrogel extracts for up to 72 h. The PCA and PC hydrogels exhibited excellent biocompatibility and low toxicity, as the relative cell viability after culture with different concentrations of the hydrogel extracts remained consistent with that of control cells (Fig. S8). Specifically, the results from the CCK-8 assays after 72 h of incubation with 40-fold dilutions of the hydrogel extracts were comparable to those of the control cells (Fig. 3b); thus, this concentration was selected for subsequent cell culture experiments. Moreover, under these coincubation conditions, cell viability remained greater than 90% for 72 h (Fig. 3a and c) and these cells retained their characteristic morphology after 24 h of incubation (Fig. 3d). Additionally, the hemocompatibility of the PCA hydrogel was assessed through incubation of red blood cells (RBCs) with serial dilutions of its extract. Notably, after being mixed with an equal volume of RBC suspension, the PCA gel extract demonstrated an excellent ability to protect erythrocytes, with a hemolysis rate less than 3% (Fig. 3e). Furthermore, OPSP at concentrations up to 3 mg/mL also exhibited a hemolysis rate below 5% (Fig. S7), further confirming the favorable hemocompatibility of the polysaccharide component. Therefore, we concluded that the successfully prepared PCA hydrogel had favorable biocompatibility.

As a potential antioxidant, the ability of PSP to scavenge radicals *in vitro* was previously established. To evaluate whether PSP could mitigate the radiation-induced accumulation of ROS associated with skin injury [38], we first assessed its scavenging capacity *in vitro* using DPPH and ABTS assays. The results showed that the DPPH and ABTS radical scavenging rates increased with increasing PSP and OPSP concentration in a dose-dependent manner (Fig. 3f and g), which confirmed its significant free radical scavenging activity.

### 3.4 Radiation protective and antioxidant capacity of the PCA hydrogel

**Fig. 4:**
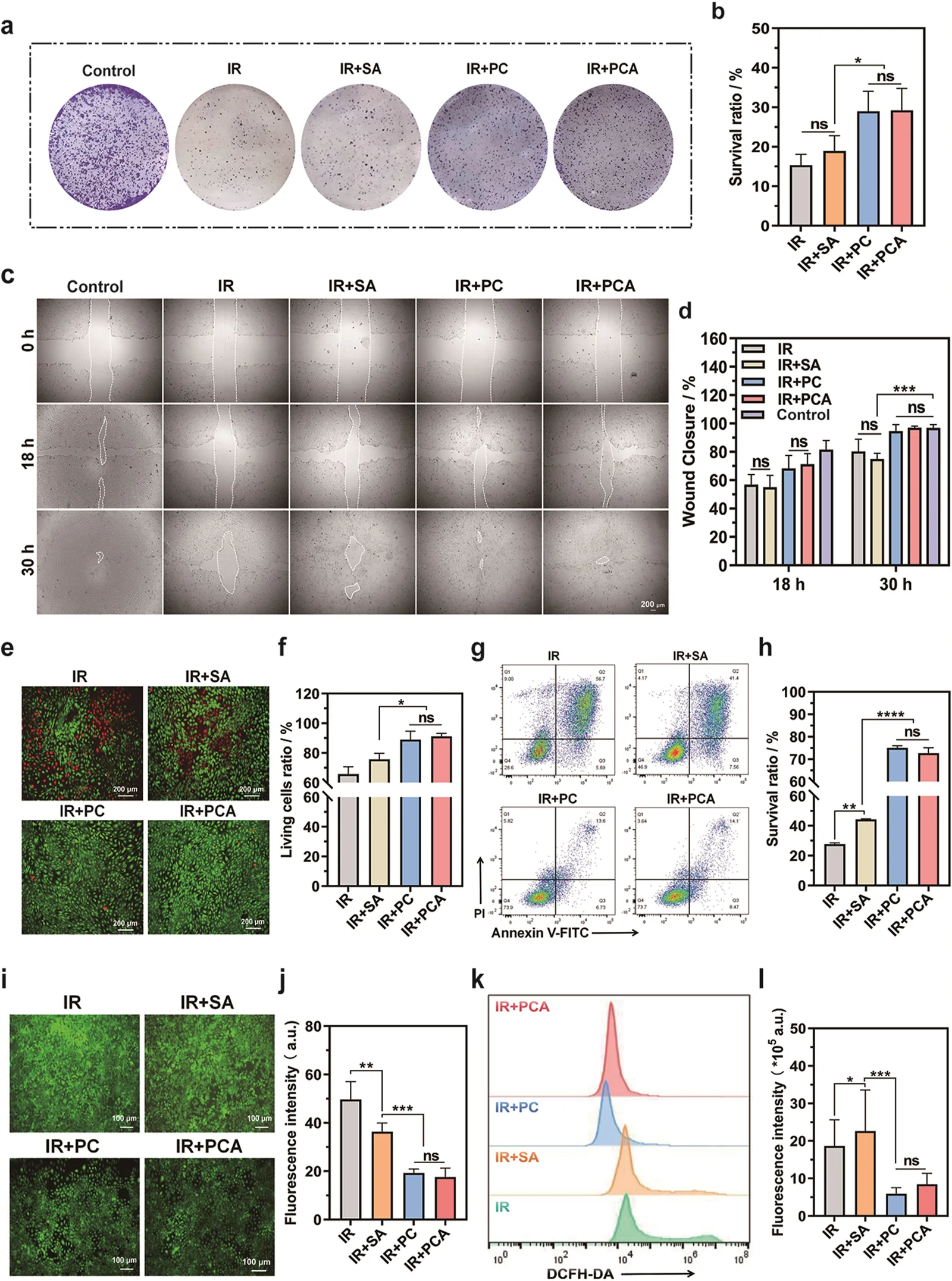
Radioprotective capacity and ROS scavenging effects of hydrogels on cells *in vitro*. (a) Representative images of cell survival colonies and (b) quantification of colony formation (n = 3). (c) Representative images of cell migration after incubation with different groups and (d) quantitative analysis of the migrated area (n = 3). Scale bars, 200 μm. (e) Representative images of Live/Dead staining of cells after different treatments and (f) quantification of live cells. Scale bars, 200 μm. (g) Flow cytometry analysis of apoptosis and (h) quantification of apoptotic rates after different treatments. (i) Representative fluorescence images of DCFH-DA staining in each group and (j) quantification of fluorescence intensity. (k) Flow cytometry histograms for ROS level and (l) statistical analysis of relative ROS fluorescence intensity. Data in (b), (d), (f), (h), (j), and (l) are presented as mean ± SD (n = 3). Statistical significance was determined using one-way ANOVA followed by Tukey's post-hoc test. *p < 0.05, **p < 0.01, ***p < 0.001, ****p < 0.0001.

To evaluate the potential radioprotective efficiency of the PCA hydrogel, we carried out a colony formation assay (Fig. 4a and b). The results demonstrated that compared with SA, PC and PCA significantly restored the colony-forming ability of cells impaired by radiation exposure. We further employed cell scratch assay to evaluate cell migration capacity after exposure to 30 Gy of X-ray irradiation. The results revealed that compared with the control treatment, the PC and PCA treatments effectively promoted cell migration over 30 h (Fig. 4c and d). Furthermore, the results of the cell viability assay confirmed that the PC and PCA gels effectively protected cells from radiation-induced cytotoxicity, more than 80% of the PC- and PCA-treated cells remained viable (Fig. 4e and f). Consistently, flow cytometry analysis validated this effect (Fig. 4g and h), revealing that the PCA gel significantly suppressed ionizing radiation-induced apoptosis. Collectively, these results confirm the multifaceted radioprotective effects of the PCA hydrogel, including increasing clonogenic survival, promoting migration, and inhibiting apoptosis.

The overproduction of ROS is not only a major contributor to cellular damage induced by ionizing radiation but also a key factor that complicates the healing process of RISI lesions compared with other types of wounds [39,40]. Intracellular ROS levels were quantified with a DCFH-DA fluorescent probe. Compared with the PC- and PCA-treated cells, the control group displayed greater fluorescence intensity after exposure to X-ray irradiation (Fig. 4i and j), indicating increased ROS levels. These results were confirmed by flow cytometry (Fig. 4k and l), demonstrating that PC and PCA significantly reduced radiation-induced ROS production and potentially protected cells from ROS-mediated oxidative damage.

### 3.5 Effect of the PCA hydrogel in the treatment of RISI

**Fig. 5:**
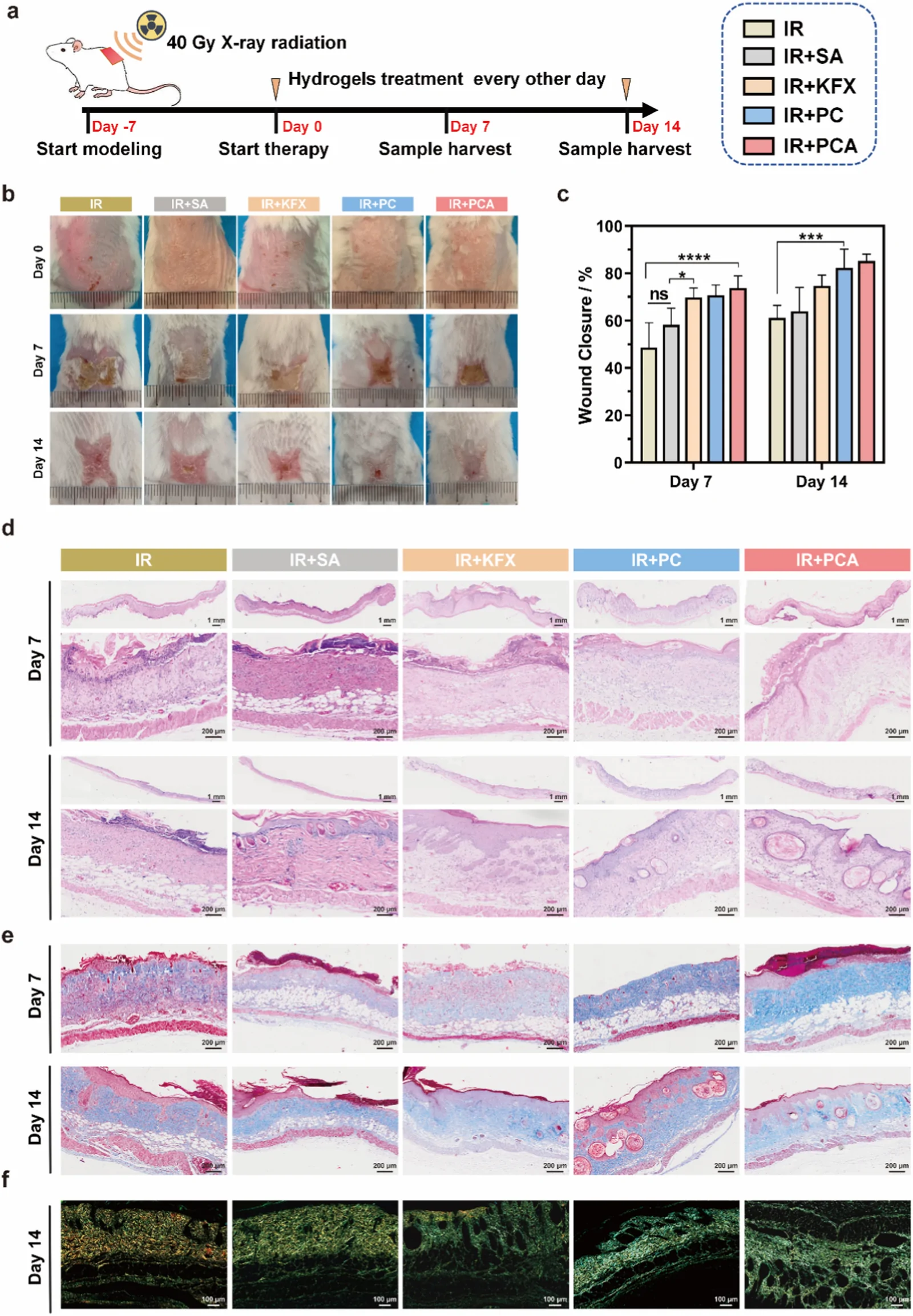
PCA hydrogel protects against irradiation-induced skin injury. (a) Schematic of the modeling and therapeutic method in a RISI model. (b) Photographs of wound healing in mice treated with different hydrogels on days 7 and 14, and (c) quantification of wound closure rates. (d-f) Representative images of H&E, Masson's trichrome and Picrosirius red staining of skin tissue sections on 7 and 14 days after different treatments. Data in (c) are presented as mean ± SD (n = 3). Statistical significance was determined using one-way ANOVA followed by Tukey's post-hoc test. *p < 0.05, ***p < 0.001, ****p < 0.0001. (For interpretation of the references to colour in this figure legend, the reader is referred to the Web version of this article.)

After confirming the promising radioprotective effects of the PSP-based hydrogel *in vitro*, we further explored the therapeutic efficacy of the PCA gel in a Balb/c mouse model of RISI. The hydrogels were topically applied one week after exposure to 40 Gy of X-ray irradiation [] every other day, and wounded skin tissue was collected for further analysis on days 7 and 14 post-treatment (Fig. 5a). Commercially available Kangfuxin Liquid was used as the positive control [31]. The results demonstrated that both the PC and PCA treatment groups exhibited significant wound closure on day 7 post-treatment, with healing rates exceeding 50% (Fig. 5b and c), which were significantly greater than those in the control and SA-treated groups. Moreover, H&E staining revealed that granulation tissue formation and re-epithelization were nearly complete in both the PC and PCA groups on day 7 after treatment (Fig. 5d). On day 14 post-treatment, compared with the other control groups, PCA group exhibited obvious skin appendage formation and significantly reduced wound bed length (Fig. S9). Histological analysis of normal skin (H&E, Masson's trichrome, and Sirius red staining) is provided in Supplementary Fig. S10 for baseline reference. Furthermore, the PCA hydrogel treatment groups displayed obvious collagen deposition that was distributed in a pattern more similar to that of normal skin (Fig. 5e). Sirius Red staining revealed pronounced type III collagen deposition in the PCA treatment group (Fig. 5f), which indicated its superior ability to promote normal tissue remodeling and regeneration after treatment. Notably, H&E staining suggested that compared with the PC group, the PCA group developed a thinner epithelial layer and more nascent skin appendages. This may be attributable to the structural modification of the PSP-based hydrogel with sodium alginate, which appears to facilitate functional skin repair.

### 3.6 Gene expression in RISI after PCA hydrogel treatment

**Fig. 6:**
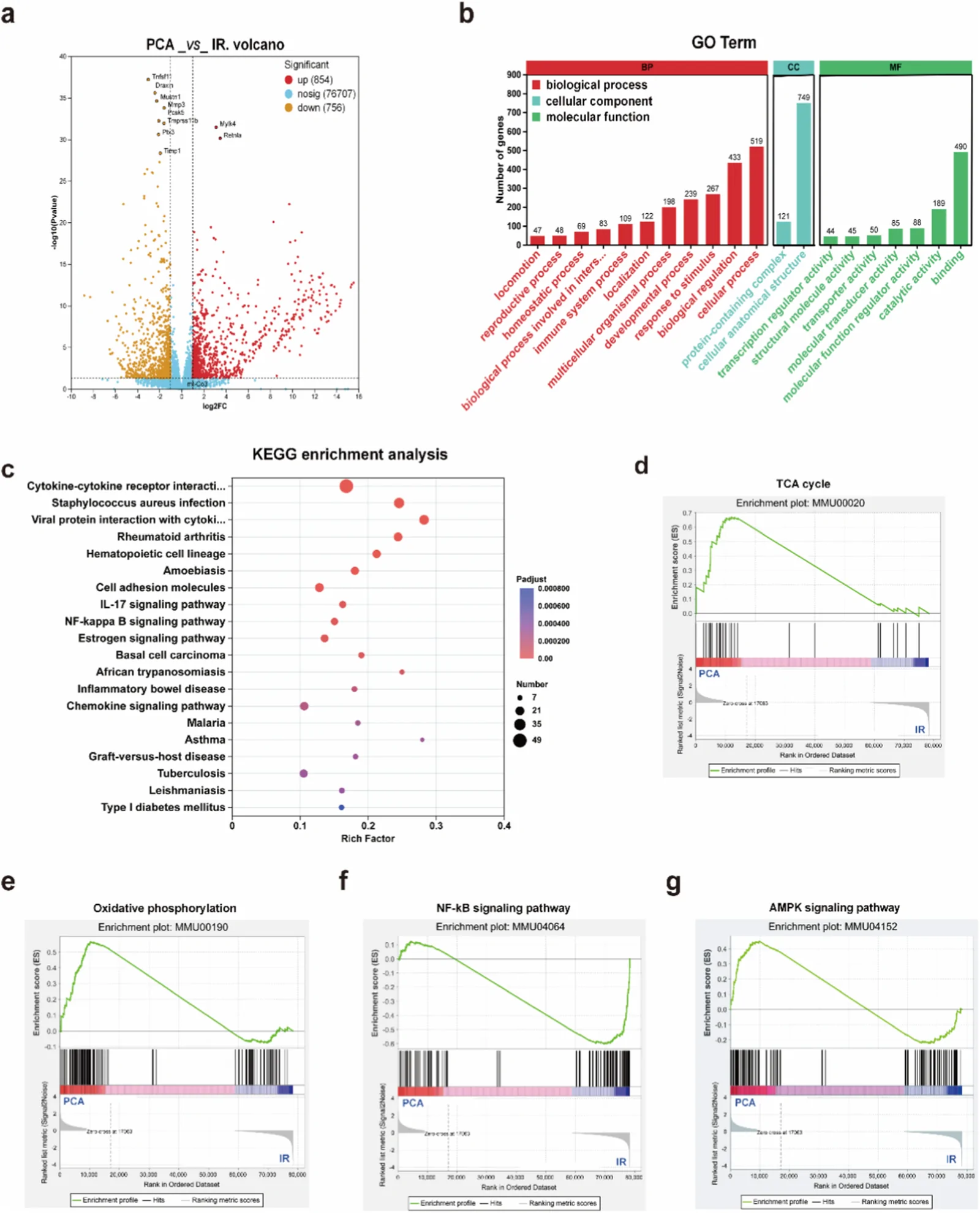
Differential gene expression analysis of RISI following PCA gel intervention. (a) Number of differentially expressed genes identified between the IR group and the PCA hydrogel group. (b) GO, and (c) KEGG enrichment analysis of differentially expressed genes between the IR group and the PCA gel group. GSEA enrichment analysis of (d) TCA cycle, (e) oxidative phosphorylation, (f) NF-κB, and (g) AMPK signal pathways between PCA and IR groups.

Building on the confirmed protective effects of the PCA hydrogel against ionizing radiation-induced damage, we subjected murine skin tissues treated with the PCA hydrogel for 14 days to transcriptomics analysis to elucidate the underlying mechanism. Skin samples from the normal skin, IR group and PCA group were collected for RNA sequencing. PCA revealed high consistency in gene expression among samples within each group (Fig. S11a). Differential gene expression analysis between the PCA group and the IR group revealed that 854 genes were upregulated and 756 genes were downregulated in the PCA group (Fig. 6a). Moreover, a heatmap revealed that the magnitude of the differences in gene expression between the normal skin group and PCA group was smaller than that between the IR group and PCA group (Fig. S11b), indicating that the transcriptional changes induced by PCA treatment were distinct from those induced by IR exposure. Gene Ontology (GO) enrichment analysis demonstrated that these differentially expressed genes were significantly associated with biological processes such as immune system processes, developmental processes, responses to stimuli, and biological regulation (Fig. 6b). Kyoto Encyclopedia of Genes and Genomes (KEGG) enrichment analysis revealed significant enrichment of multiple pathways, including the IL-17, NF-κB, and chemokine signaling pathways (Fig. 6c), which is consistent with the various pathological features of radiation dermatitis. Reactome annotation analysis revealed alterations mainly in terms of developmental biology and keratinization for epidermal regeneration, the immune system for inflammation resolution, and lipid metabolism and global metabolism for nutrient and energy supply (Fig. S11c).

Furthermore, gene set enrichment analysis (GSEA) was carried out to analyze the activated and suppressed signaling pathways (Fig. 6d–g). The results revealed that oxidative phosphorylation (OXPHOS) and tricarboxylic acid (TCA) cycle were enriched in the PCA group. Additionally, compared with the IR group, the PCA-treated group exhibited significant activation of AMPK signaling and suppression of NF-κB signaling. On the basis of these findings, we constructed a heatmap to analyze the expression levels of the corresponding clustered genes. With respect to the TCA cycle, the genes Dlat, Idh3g, and Cs were significantly upregulated in the PCA gel-treated group (Fig. S11d), which promotes an increase in mitochondrial energy metabolism and provides ATP for the proliferation, differentiation, and functional recovery of skin cells during the repair of RISI lesions. With respect to OXPHOS enrichment, the genes Wnt9a, Dbt, Ndufa9, and Mmp14 were upregulated in the PCA gel-treated group (Fig. S11e), demonstrating that the PCA hydrogel activated the mitochondrial respiratory chain to ameliorate radiation-induced mitochondrial dysfunction. This can also reduce excessive ROS production and alleviate oxidative stress-mediated tissue injury [41,42]. Further analysis of signaling pathways (Fig. S11f) revealed that the genes Relb, Tnfsf14, Bcl2l1, and Card14, which are related to the NF-κB signaling pathway, were downregulated [43], whereas the genes Atp5f1e, mt-Co3, Uqcr11, and Atp5pd, which are involved in the AMPK signaling pathway, were upregulated (Fig. S11g). This dual regulatory mode indicated that the PCA gel regulated the inflammatory response and metabolic homeostasis, including the suppression of NF-κB signaling to reduce the excessive secretion of proinflammatory cytokines and the activation of AMPK signaling to promote energy metabolism and cell survival.

### 3.7 The PCA hydrogel alleviates RISI owing to its antioxidant, anti-inflammatory effects and promotion of macrophage polarization

The transcriptomic data indicated that the PCA hydrogel alleviates RISI by modulating inflammatory responses and restoring metabolic homeostasis. Given the established importance of macrophages in wound inflammation and the previous observations of their pronounced phenotypic and functional impairment in RISI models [7,44], we focused our investigation on macrophage function.

**Fig. 7:**
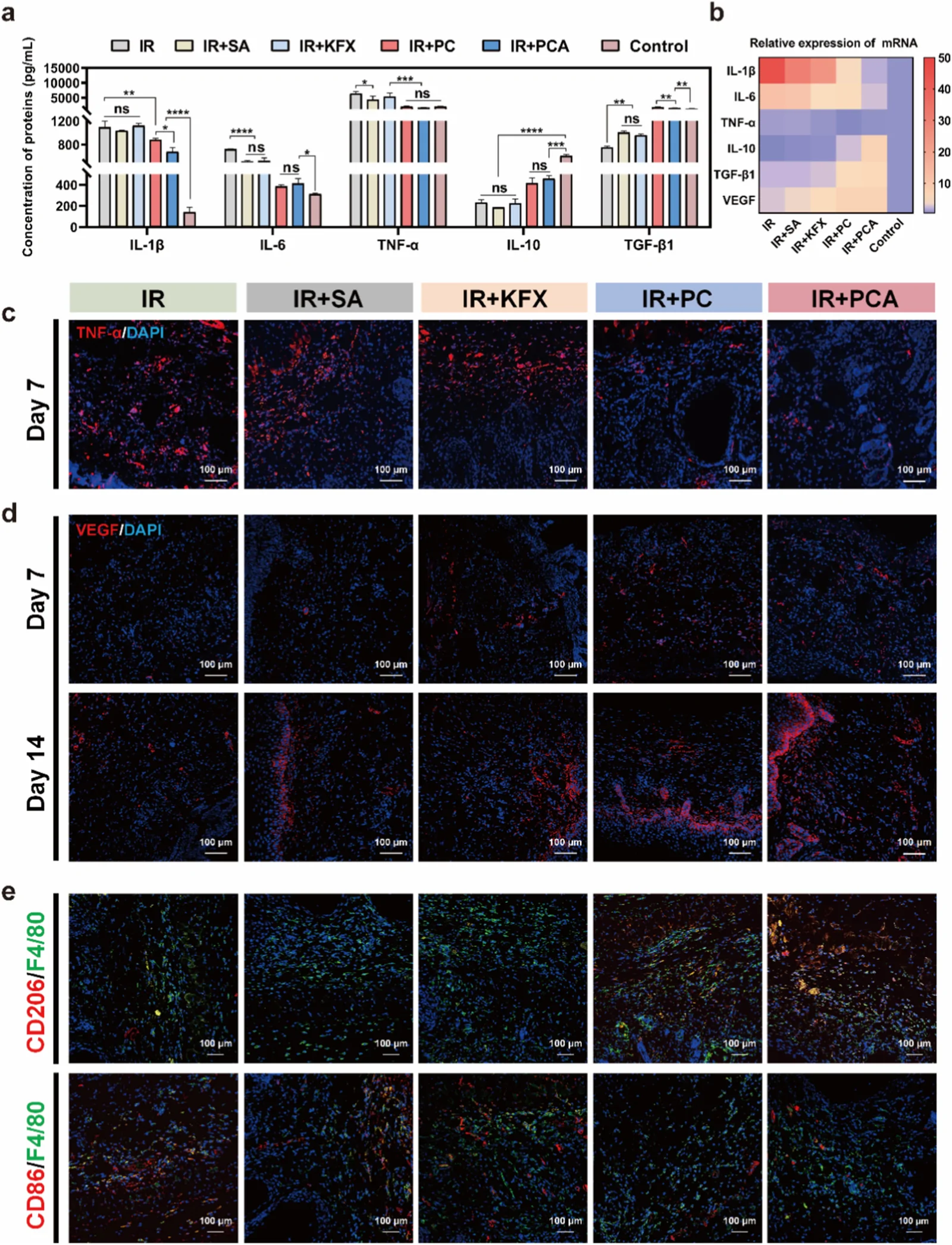
PCA hydrogel inhibits irradiation-induced oxidative stress, inflammation, and macrophage polarization. (a) ELISA and (b) RT-qPCR analysis of pro-inflammatory (IL-6, IL-1β, and TNF-α) and anti-inflammatory (IL-10, TGF-β1) cytokine levels in skin samples. (c) Representative of immunofluorescence staining images of TNF-α (TNF-α, red; DAPI, blue) on day 7. Scale bar, 100 μm. (d) Immunofluorescence staining of VEGF (VEGF, red; DAPI, blue) for wounds on days 7 and 14. Scale bar, 100 μm. (e) Immunofluorescence staining of CD86 (CD86, red; F4/80, green) and CD206 (CD206, red; F4/80, green) for wounds on day 14. Scale bar, 100 μm. Data in (a) are presented as mean ± SD (n = 3). Statistical significance was determined using one-way ANOVA followed by Tukey's post-hoc test. *p < 0.05, **p < 0.01, ***p < 0.001, ****p < 0.0001. (For interpretation of the references to colour in this figure legend, the reader is referred to the Web version of this article.)

On the basis of previous findings, we proposed that the PCA hydrogel could mitigate excessive oxidative stress damage caused by radiation exposure. We therefore analyzed tissue inflammation via ELISA (Fig. 7a) and RT-qPCR (Fig. 7b). The results demonstrated that PCA hydrogel treatment significantly reduced the expression of proinflammatory cytokines (IL-1β, IL-6, and TNF-α) at both the gene and protein levels while decreasing the expression of anti-inflammatory cytokines (IL-10 and TGF-β1) in wound tissues. Immunofluorescence staining for TNF-α confirmed these results (Fig. 7c and S13a), with the PCA-treated group showing lower TNF-α expression levels than the IR treatment group. Immunofluorescence staining of normal skin for TNF-α, VEGF, CD86/F4/80, and CD206/F4/80 is provided in Supplementary Fig. S12 for baseline reference. Furthermore, vascular endothelial growth factor (VEGF) was upregulated by PCA hydrogel treatment (Fig. 7d and S13b), which correlates with the shift to an anti-inflammatory cytokine profile, demonstrating the ability of the PCA hydrogel to act as an immune switch and promote healing. As VEGF is a hallmark of M2-like macrophage polarization, the upregulation of this factor further supports the transition to a tissue-reparative state, which is essential for subsequent proliferation and remodeling in the treatment of RISI [45]. Therefore, we performed immunofluorescence staining to analyze the ratios of CD86 and CD206 expression to F4/80. PCA gel treatment decreased the CD86/F4/80 ratio but increased the CD206/F4/80 ratio (Fig. 7e and S13c), indicating that the transition of injured tissue from the proinflammatory phase to the anti-inflammatory phase was mediated by modulating macrophage polarization, thereby alleviating excessive inflammation in the wound [46]. These results demonstrated that the PCA hydrogel mitigated RISI by reducing oxidative stress and promoting the polarization of macrophages toward a reparative phenotype.

### 3.8 The PCA hydrogel protects macrophages from radiation injury by restoring mitochondrial homeostasis

**Fig. 8:**
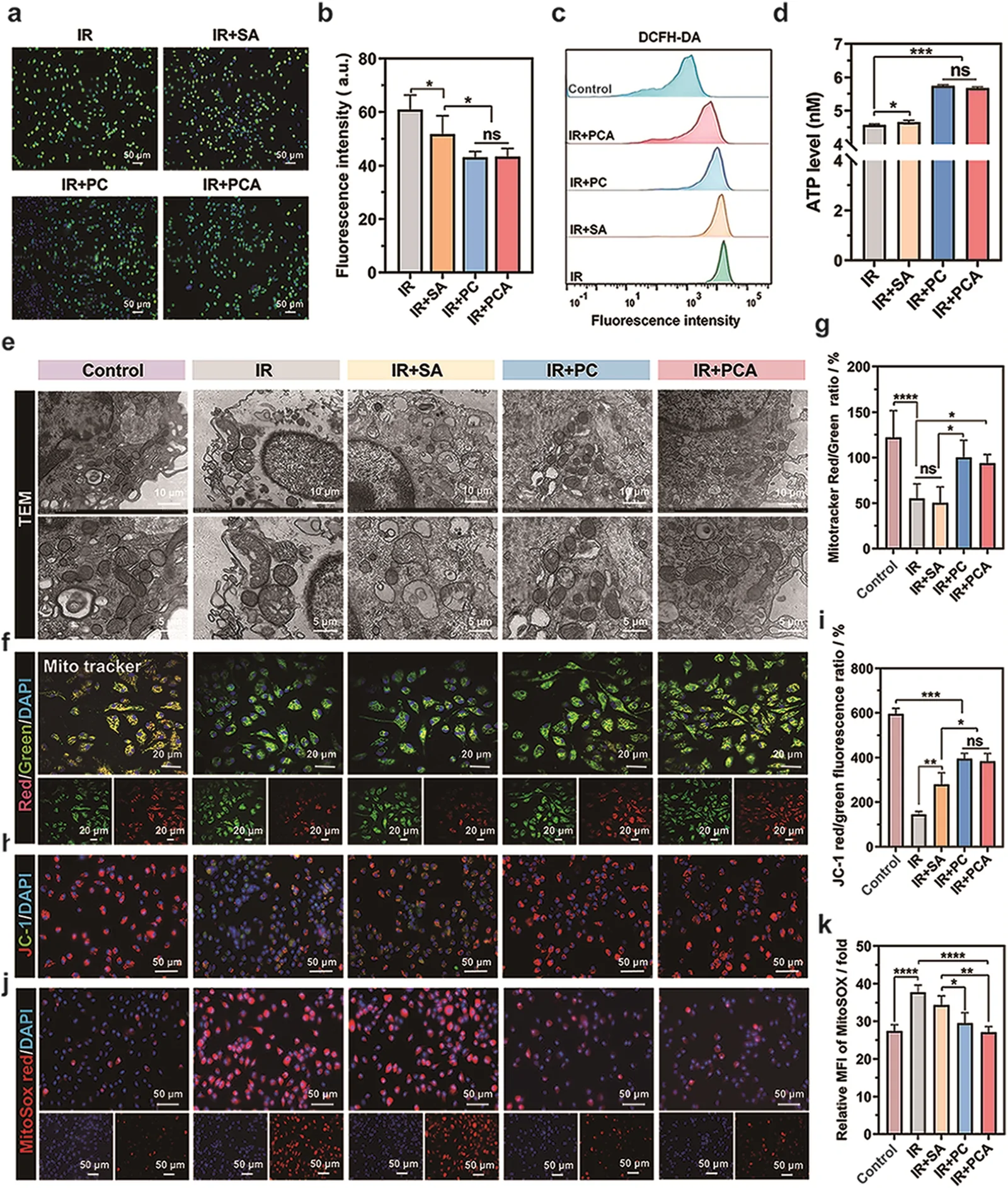
PCA hydrogel alleviates excessive oxidative stress and ameliorates mitochondrial dysfunction in irradiated macrophages *in vitro*. (a) Representative images of DCFH-DA staining for ROS and (b) corresponding fluorescence intensity quantification. Scale bar, 50 μm. (c) Flow cytometry analysis of ROS levels in PMs after different treatment. (d) ATP expression levels in different groups. (e) Representative images of TEM of mitochondrial ultrastructure after different treatment. Scale bar, 10 μm. (f) Representative images and (g) quantification of dysfunctional mitochondria (MitoTracker Red/MitoTracker Green ratio) in PMs. Scale bar, 20 μm. (h) Representative images of JC-1 staining (monomers, green; oligomers, red) and (j) MitoSOX™ Red staining for mtROS, and (i, k) corresponding fluorescence intensity quantification in PMs following different treatments. Scale bar, 50 μm. Data in (b), (d), (g), (i), and (k) are presented as mean ± SD (n = 3). Statistical significance was determined using one-way ANOVA followed by Tukey's post-hoc test. *p < 0.05, **p < 0.01, ***p < 0.001, ****p < 0.0001. (For interpretation of the references to colour in this figure legend, the reader is referred to the Web version of this article.)

On the basis of the *in vivo* and transcriptomics results, we hypothesized that the PCA hydrogel exerts immune and inflammatory regulatory effects by modulating macrophage function. Therefore, we established an *in vitro* model of X-ray radiation-induced macrophage injury to evaluate the radioprotective effect and related mechanisms of the PCA gel. The results of the CCK-8 assay revealed that 4 Gy of irradiation-maintained cell viability (Fig. S14). Under these conditions, the ability of the PCA hydrogel extract to promote the phagocytic capacity of macrophages was greater than that of the other groups (Fig. S15), suggesting that the PCA gel extract protected macrophage function after irradiation. We subsequently evaluated the ability of the PCA hydrogel to scavenge intracellular ROS using the fluorescent probe DCFH-DA. Immunofluorescence staining (Fig. 8a and b) and flow cytometry analysis (Fig. 8c and S16) demonstrated that PCA treatment significantly reduced the ROS levels in irradiated PMs, demonstrating its protective effect against radiation-induced oxidative injury. Moreover, we observed that the PCA hydrogel protected macrophages from ATP depletion caused by radiation (Fig. 8d). Studies have demonstrated that mitochondria are the energy factories of cells and sustain cellular functions via ATP production. However, dysfunctional mitochondria produce excessive amounts of ROS, which intensifies injury via a vicious cycle [47]. Given that the PCA hydrogel can scavenge ROS and maintain ATP levels, we hypothesized that it could protect macrophages from radiation-induced mitochondrial oxidative stress and dysfunction.

Furthermore, TEM was performed to evaluate the changes in mitochondrial structure in macrophages after irradiation. The results revealed that the mitochondrial ultrastructure was severely damaged by radiation (Fig. 8e), which manifested mainly as membrane rupture, cristae loss, and swelling. In contrast, PCA maintained the integrity of the double membrane and cristae morphology, which demonstrated their ability to protect the mitochondrial structure. To assess whether mitochondrial dysfunction occurred after post-irradiation, we performed immunofluorescence staining using MitoTracker Red (a marker of functional mitochondria) and MitoTracker Green (a general mitochondrial marker). The results revealed that radiation significantly reduced the fluorescence ratio of MitoTracker Red to MitoTracker Green (Fig. 8f and g), which indicated that functional mitochondria were depleted after radiation. In contrast, this ratio was maintained in the PC and PCA treatment groups, indicating that these hydrogels alleviate mitochondrial membrane potential depletion to preserve mitochondrial function, thereby maintaining energy and redox homeostasis in irradiated cells [48]. Consistent with these results, JC-1 staining confirmed that compared with that in the other groups, the red/green fluorescence ratio was greater in the PCA treatment group (Fig. 8h and i), indicating that the mitochondrial membrane potential stabilized. Given that a stable mitochondrial membrane potential is crucial for maintaining normal mitochondrial function and that disruption of this potential leads to electron transport chain leakage and increased ROS levels [49], we measured the level of mitochondrial reactive oxygen species (mtROS). MitoSOX Red staining confirmed that PCA treatment effectively scavenged excess mtROS after irradiation (Fig. 8j and k). These results demonstrated that the PCA gel disrupted the cycle of ROS and mitochondrial damage, offering direct evidence for mitochondrial protection at the level of redox homeostasis [50].

To further verify the causal role of mitochondrial homeostasis in PCA-mediated immunomodulation, we performed a reverse intervention experiment using rotenone, a specific mitochondrial complex I inhibitor. We first confirmed that 1 μM rotenone did not affect macrophage viability (Fig. S17 and S18a, 18b). JC-1 staining revealed that PCA significantly restored the radiation-induced loss of mitochondrial membrane potential, whereas rotenone co-treatment effectively blocked this protective effect (Fig. S18c and 18d). Consistently, rotenone reversed PCA-induced M2 polarization, as evidenced by decreased CD206 and ARG1 expression, increased CD86 and iNOS expression (Fig. S19a–f), and reduced IL-10 secretion (Fig. S19g). These results demonstrate that mitochondrial homeostasis is an upstream causal prerequisite for PCA-mediated immunomodulation.

### 3.9 The PCA hydrogel modulates mitochondrial metabolism in irradiated macrophages

**Fig. 9:**
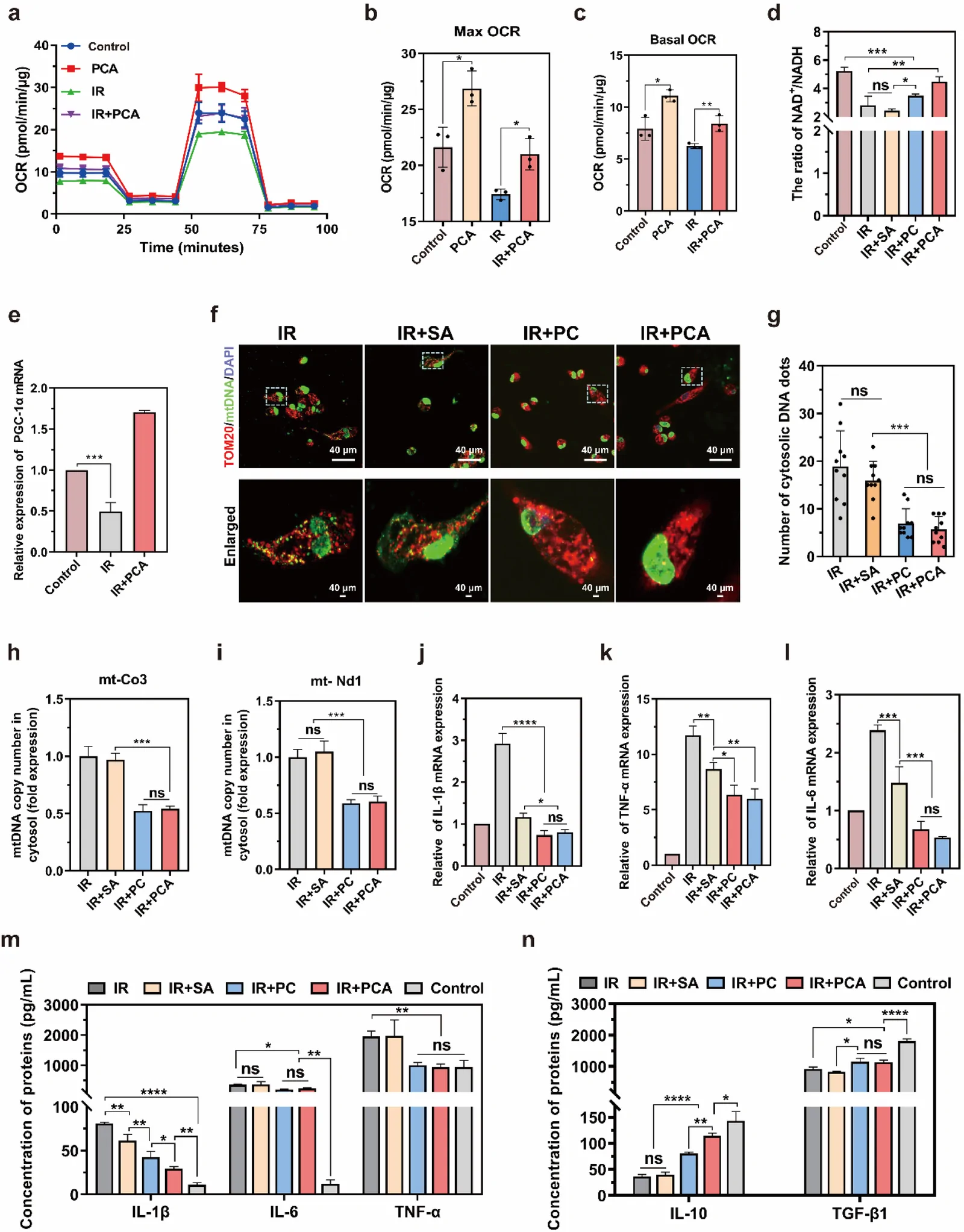
PCA hydrogel ameliorates radiation-induced mitochondrial dysfunction via metabolic and inflammatory regulation in macrophages. (a) Oxygen consumption rates (OCRs) measured by Seahorse XFe96 Analyzer, with quantification of (b) maximal OCR and (c) basal OCR in different treatment groups. (d) Relative NAD^+^/NADH ratio in macrophages after different treatment. (e) RT-qPCR analysis of PGC-1α mRNA expression. (f) Representative immunofluorescence images of mtDNA staining (mtDNA, green; TOM20, red), and (g) corresponding quantification. (h, i) qPCR analysis of cytosolic mitochondrial DNA (mt-CO3, mt-Nd1). (j-l) RT-qPCR analysis of pro-inflammatory cytokine expression levels. (m) ELISA analysis of pro-inflammatory and (n) anti-inflammatory cytokine protein levels. All data are presented as mean ± SD (n = 3). Statistical significance was determined using one-way ANOVA followed by Tukey's post-hoc test. *p < 0.05, **p < 0.01, ***p < 0.001, ****p < 0.0001. (For interpretation of the references to colour in this figure legend, the reader is referred to the Web version of this article.)

On the basis of the findings that the PCA hydrogel could maintain the structural integrity of mitochondria and reduce mitochondrial oxidative stress, we conducted Seahorse analysis to assess mitochondrial oxidative phosphorylation. The results indicated that radiation impaired oxidative phosphorylation, leading to a reduction in the maximum and basal oxygen consumption rates (OCRs) (Fig. 9a). However, PCA treatment protected the respiratory function of mitochondria and maintained the OCR value near baseline after radiation (Fig. 9b and c). Conversely, the ratio of NAD^+^/NADH, which reflects the redox state within cells, significantly decreased, but PCA treatment effectively reversed this trend by significantly increasing the NAD^+^/NADH ratio (Fig. 9d). Moreover, RT-qPCR results showed that PCA treatment significantly upregulated the expression of PGC-1α (a key regulator of mitochondrial biogenesis), which further supported the recovery of mitochondrial function (Fig. 9e). Overall, these results indicated that PCA treatment could reshape cellular energy metabolism after radiation-induced damage by improving mitochondrial function and the homeostasis of core metabolites.

As a damage-associated molecular pattern, the release of mitochondrial DNA (mtDNA) is a key upstream event that activates the inflammatory response [51]. After confirming that the PCA hydrogel could effectively maintain mitochondrial homeostasis, we further evaluated the leakage of mtDNA into the cytoplasm. Immunofluorescence staining revealed that mtDNA (green) was abnormally dispersed from the mitochondrial network (TOM20, red) after radiation, while PCA treatment significantly maintained the colocalization of these molecules (Fig. 9f and g). These morphological observations demonstrated the protective effect of PCA on the spatial localization of mtDNA. Consistently, qPCR of the cytoplasmic fraction revealed that PCA treatment significantly reduced the levels of free mtDNA (mt-CO3 and mt-Nd1) in irradiated cells (Fig. 9h and i). Collectively, these results revealed that the PCA hydrogel maintained mtDNA stability and prevented abnormal mtDNA leakage, thereby establishing a crucial genetic foundation for the recovery of mitochondrial energy metabolism. Given the established link between mitochondrial dysfunction and inflammation, which is often mediated by mtDNA leakage [52], we further evaluated the effect of the PCA hydrogel on the immune phenotype by analyzing the production of cytokines. RT-qPCR revealed that PCA treatment inhibited the transcription of key proinflammatory cytokines (Fig. 9j–l). This anti-inflammatory effect was confirmed at the protein level by ELISA, which revealed that PCA not only inhibited the secretion of proinflammatory cytokines but also promoted the release of anti-inflammatory cytokines (Fig. 9m and n). In conclusion, these data indicated that PCA corrected the inflammatory imbalance caused by radiation and promoted the formation of an immune microenvironment conducive to repair.

In summary, our data demonstrated that the PCA hydrogel preserved mitochondrial structural and functional integrity in irradiated macrophages to restore mitochondrial homeostasis. This mitochondrial-centric effect provides fundamental support for its subsequent immunomodulatory efficacy, driving the polarization of macrophages toward a proreparative phenotype for inflammation resolution.

## 4 Discussion

RISI is a major complication of tumor radiotherapy, the refractory healing mechanisms of which involve multiple factors, such as persistent DNA damage, excessive oxidative stress, and dysregulated cellular metabolism [53]. Current therapeutic methods focus mainly on symptomatic treatment to accelerate wound healing, but such passive strategies are inadequate for addressing the persistent cellular dysfunction triggered by ionizing radiation, particularly the overproduction of ROS by damaged mitochondria, which exacerbates tissue injury. Consequently, interventions that can not only promote repair but also actively regulate the dysregulated inflammatory response are urgently needed.

Our study demonstrated that the PCA hydrogel not only effectively protected against radiation-induced damage *in vitro* and *in vivo* but also, more importantly, restored mitochondrial function in macrophages by alleviating oxidative stress and restoring energy metabolism, thereby directly reprogramming their inflammatory phenotype. This mitochondrion-centric active intervention strategy offers a novel therapeutic approach to address the dual challenge of tissue repair and immunometabolism dysregulation in the context of radiodermatitis.

Previous work indicated that ionizing radiation impaired macrophage function, leading to inflammatory imbalance and defective clearance of senescent cells [7,54]; however, the underlying mechanisms remained unclear. Here, we demonstrated that radiation-induced mitochondrial dysfunction represented a key pathological event that aggravated oxidative stress and promoted the leakage of mitochondrial DNA (mtDNA), thereby sustaining inflammatory responses [55]. The PCA hydrogel counteracted this cascade by scavenging ROS, preserving ATP production, restoring mitochondrial ultrastructure and membrane potential, and improving respiratory function, thereby collectively re-established mitochondrial homeostasis. Notably, PCA markedly reduced cytosolic mtDNA release, effectively interrupting the vicious cycle of mitochondrial damage, oxidative stress, and inflammation. Mechanistically, PCA could activate the AMPK signaling pathway to restore mitochondrial fitness while suppressing the NF-κB pathway to inhibit inflammatory responses. These effects polarized macrophages toward an M2 repair phenotype, ultimately restoring immune homeostasis and facilitating tissue repair.

In recent years, research on biomaterials for RISI treatment has attracted significant attention. However, although biomaterials such as phycocyanin [5], short peptides [4], and selenomelanin [56] have demonstrated promising therapeutic potential, novel strategies that can actively regulate the pathological process of RISI and correct cellular and molecular dysfunction are being explored in both clinical practice and fundamental studies. Kangfuxin Liquid, as a traditional Chinese medicine that has been clinically explored for the treatment of RISI, provides a meaningful positive control for evaluating our PCA hydrogel [31]. This finding indicates that even bioactive components proven effective in skin wound repair may require targeted optimization in certain aspects, such as the route of administration, formulation, or therapeutic time window, when applied for RISI treatment. The PCA hydrogel we developed in this study represents an active regulatory strategy. It directly acts on and repairs the structure and function of macrophage mitochondria and inhibits mtDNA leakage, thereby polarizing macrophages toward a reparative phenotype for RISI repair.

Admittedly, the *in vitro* experiments were performed using hydrogel extracts, which may not fully reflect the combined chemical and physical contributions of the intact hydrogel matrix. Nonetheless, many challenges need to be overcome before the clinical application of the PCA hydrogel. In addition to establishing strict evaluation criteria for the safety and efficacy of each component, developing efficient and scalable extraction and standardized purification processes remains a key issue that urgently needs to be addressed. More importantly, the underlying mechanism of PSP in this study was merely speculated based on transcriptomic analysis. Further molecular biological investigations are required to deeply explore the AMPK and NF-κB signaling pathways and clarify the exact mechanism of PSP action. Meanwhile, further studies incorporating primary macrophage sorting from RISI models are required to elucidate the regulatory effects of PSP on macrophage function at the wound site.

## 5 Conclusion

In conclusion, we designed a multifunctional PCA hydrogel with excellent protective and reparative effects against radiation-induced skin injury. This hydrogel effectively alleviates oxidative stress and excessive inflammatory responses, and exerts beneficial regulatory effects on macrophage biological function. Currently, effective strategies capable of regulating macrophage mitochondrial homeostasis in RISI treatment are still lacking. The macrophage mitochondrial homeostasis regulatory strategy proposed in this study not only provides a new option for RISI treatment but also offers insights into the design of regenerative materials for inflammatory tissue injury, highlighting its favorable translational potential.

## CRediT authorship contribution statement

**Wenfeng Li:** Data curation, Formal analysis, Funding acquisition, Investigation, Writing – original draft. **Yawei Wang:** Data curation, Formal analysis, Validation. **Yang Xu:** Data curation, Formal analysis. **Ziyan Zhou:** Data curation, Investigation. **Yang Wang:** Formal analysis, Validation. **Quanying Liu:** Data curation, Investigation. **Chunmeng Shi:** Funding acquisition, Project administration, Supervision, Writing – review & editing.

## Declaration of competing interest

The authors declare that they have no known competing financial interests or personal relationships that could have appeared to influence the work reported in this paper.

## Data Availability

Data will be made available on request.
