# Supplementary material for: Polygonatum sibiricum polysaccharide-based hydrogels regulate macrophage mitochondrial homeostasis for the treatment of radiodermatitis
Source: Mater Today Bio. 2026 Aug 4;40:103520. doi: 10.1016/j.mtbio.2026.103520 (PMC13488034; doi:10.1016/j.mtbio.2026.103520)
Supplement: Supplementary file 1 [file mmc1.docx]

***Polygonatum sibiricum* polysaccharide-based hydrogels regulate macrophage mitochondrial homeostasis for the treatment of radiodermatitis**

# Extended data figures and tables

**1. GPC analysis.**

The molecular weight of Qcs was determined by gel permeation chromatography (GPC) using an Agilent 1260 Infinity II system equipped with a refractive index detector and TSKgel GMPWxl columns (Tosoh Bioscience). The sample was dissolved in 0.1 M NaNO₃ solution at 1.0 mg/mL and filtered through a 0.45 μm membrane prior to injection. The flow rate was 0.6 mL/min, the column temperature was 40 °C, and the injection volume was 10.0 μL. Glucan standards were used for calibration. Data were analyzed using Cirrus GPC software. The calculated Mn, Mw, and Mp values are summarized in Table S1, and the GPC chromatogram and molecular weight distribution curve are shown in Fig. S1.

**Fig.S1 GPC analysis of QCS.** a. GPC chromatograms. b. Molecular weight distribution plot of QCS.

**Table S1** Molecular weight parameters of Qcs determined by GPC

| Parameter | Mn | Mw | Mp | Mz | PDI (Mw/Mn) |
| --- | --- | --- | --- | --- | --- |
| Value (g/mol) | 1890 | 1991 | 1963 | 2101 | 1.05 |

**2. Monosaccharide composition analysis**

The monosaccharide composition of the polysaccharide was determined by 1-phenyl-3-methyl-5-pyrazolinone (PMP) derivatization, which is applicable for reduced monosaccharides. Briefly, the PSP and OPSP powders were hydrolyzed with trifluoroacetic acid (TFA), derivatized with a PMP precolumn, and then analyzed by HPLC (Agilent 1200, Germany) to determine their monosaccharide compositions. The following 14 monosaccharide standards were used: L‑guluronic acid (Glu), mannuronic acid (ManA), mannose (Man), glucosamine (GlcN), ribose (Rib), rhamnose (Rham), glucuronic acid (GlcUA), galacturonic acid (GalUA), galactosamine (GalN), glucose (Glc), galactose (Gal), xylose (Xyl), arabinose (Ara) and fucose (Fuc). First, 15 mg of PSP or OPSP powder was added to 5 mL of 2 mol/L TFA, after which the tube was filled with nitrogen before hydrolysis in a 110 °C oven for 2 h. After the samples had completely cooled, 1 mL of the hydrolysate was mixed with an equal volume of methanol and evaporated to dryness under a stream of nitrogen at 70 °C. The methanol addition and N_2_ drying protocol was repeated twice to remove all residual TFA. The residue was subsequently completely dissolved in 1 mL of 0.3 mol/L NaOH solution for subsequent derivate analysis after dilution. In the second step, which involved monosaccharide derivatization, 400 μL of the standard mixture or polysaccharide solution was transferred to a test tube, an equal volume of PMP was added, and the mixture was heated at 70 °C for 2 h. After cooling to room temperature, 400 μL of 0.3 mol/L HCl was added to adjust the pH to 6~7. Next, an equal volume of chloroform was added, the solution was vortexed and allowed to stand for 10 min, and the chloroform was discarded; this extraction process was repeated twice. The resulting aqueous phase was filtered through a 0.45 μm membrane prior to HPLC analysis. Finally, HPLC analysis was performed with a C18 column (250 mm **×** 4.6 mm, 5 μm) at 30 °C with detection at 245 nm. The mobile phase consisted of 100 mM sodium phosphate buffer (pH 6.7) and acetonitrile. The flow rate was 1 mL/min, and the injection volume was 5 μL.

**3. Rotenone treatment and experimental design**

To investigate whether mitochondrial homeostasis serves as an upstream causal prerequisite for the immunomodulatory effects of PCA, we performed a reverse intervention experiment using rotenone, a specific mitochondrial complex I inhibitor.

First, we determined the optimal working concentration of rotenone in macrophages. Cells were seeded in 96-well plates at a density of 1 × 10⁴ cells per well and treated with different concentrations of rotenone (0.1, 0.5, 1.0, 2.5, and 5.0 μM) for 24 h and 48 h. Cell viability was assessed using the CCK-8 assay (Beyotime, China) according to the manufacturer’s instructions. Based on the dose‑response results, 1 μM rotenone was selected for subsequent experiments, as this concentration effectively inhibited mitochondrial complex I without causing significant cytotoxicity.

For the mechanistic evaluation, macrophages were incubated with PCA for 24 h, followed by irradiation. Immediately after irradiation, rotenone (1 μM) was added to the designated groups. Cells were collected at 12 h and 24 h post‑irradiation for further analyses. The experimental groups included: (1) Control (untreated), (2) IR (irradiation alone), (3) IR + PCA, and (4) IR + PCA + Rotenone.

Mitochondrial membrane potential (ΔΨm) was further evaluated using the JC‑1 fluorescent probe (Beyotime, China). Briefly, at 12 h and 24 h post‑irradiation, macrophages were incubated with JC‑1 staining solution (5 μg/mL) at 37 °C for 20 min in the dark. After washing twice with JC‑1 buffer, cells were immediately observed under a fluorescence microscope (Olympus, Japan). JC‑1 exists as red‑fluorescent aggregates (J‑aggregates) at high membrane potential and as green‑fluorescent monomers (J‑monomers) at low membrane potential. The red/green fluorescence intensity ratio was quantified using ImageJ software to reflect changes in mitochondrial membrane potential. At least three randomly selected fields per group were analyzed.

To evaluate macrophage polarization, cells were seeded on glass coverslips in 24‑well plates and subjected to the aforementioned treatments. At 24 h post‑irradiation, cells were fixed with 4% paraformaldehyde for 15 min at room temperature, permeabilized with 0.25% Triton X‑100 for 10 min, and blocked with 5% bovine serum albumin (BSA) for 1 h at 37 °C. Cells were then incubated overnight at 4 °C with primary antibodies against CD86 (1:200, 13395-1-AP), iNOS (1:400, ab178945), CD206 (1:200, ab64693), and ARG1 (1:400, 93668s). After washing with PBST, cells were incubated with Alexa Fluor‑conjugated secondary antibodies (1:500, Beyotime) for 1 h at 37 °C in the dark. Nuclei were counterstained with DAPI for 5 min. Coverslips were mounted on glass slides and images were captured using an Olympus FV3000 confocal laser scanning microscope (Olympus, Japan). For each group, at least three randomly selected fields were acquired, and the percentage of positive cells for each marker was quantified using ImageJ software. To evaluate macrophage polarization and the associated anti‑inflammatory response, IL‑10 levels in cell culture supernatants were measured by ELISA at 24 h post‑irradiation (CUSABIO, China).

**Fig.S2** Representative SEM images and the corresponding diameter distribution profiles of different concentrations of OPSP solution mixed with Qcs into hydrogels.

**Fig.S3** Rheological behaviors of 2% (w/v) OPSP/Qcs mixtures at different volume/mass ratios. (a) Gelation time; (b) Viscosity.

**Fig.S4** The injectability demonstration of PCA hydrogel using syringes of different sizes.

**Fig.S5** Confocal 3D reconstruction images of PC and PCA hydrogels. The 3D projection images show the continuous and interconnected network structure of the PC hydrogel before (left) and after (right) incorporation of the SA‑Ca²⁺ network. Scale bar = 100 μm.

**Fig.S6** In vitro cumulative release profiles of FITC-OPSP from PC and PCA hydrogels in PBS (pH 7.4) at 37 °C. Data are presented as mean ± SD (n = 3).

**Fig.S7** Hemolysis rates of red blood cells treated with OPSP at various concentrations. Saline and distilled water were used as negative and positive controls, respectively. Data are presented as mean ± SD (n = 3). Statistical significance was determined using one‑way ANOVA followed by Tukey's post‑hoc test. ***p < 0.001.

**Fig.S8** CCK‑8 assay of hydrogels. Hydrogels (200 μL) were immersed in 30 mL of culture medium and serially diluted before incubation with cells. Cell viability was assessed. Data are presented as mean ± SD (n = 3). Statistical significance was determined using one‑way ANOVA followed by Tukey's post‑hoc test. *p < 0.05.

**Fig.S9** Quantitative analysis of the wound bed length of wound area on days 7 and 14 in H&E staining. n = 3. Data are presented as mean ± SD. Statistical significance was determined using one‑way ANOVA followed by Tukey's post‑hoc test. *p < 0.05, **p < 0.01.

**Fig.S10** Histological analysis of normal mouse skin. (a) H&E staining; (b) Masson's trichrome staining; (c) Sirius red staining. Scale bars: (a) 1 mm; 100 μm, (b) and (c) 100 μm.

**Fig.S11** Differential gene expression analysis of RISI following PCA gel intervention. (a) Gene correlation analysis among Control, IR, and PCA hydrogel groups. (b) Clustering heatmap of DEGs between the PCA hydrogel and IR groups. (c) Reactome enrichment analysis of differentially expressed genes between the IR group and the PCA gel group. (d-g) GSEA gene enrichment heatmaps of the TCA cycle, oxidative phosphorylation, NF-κB and AMPK signaling pathways in the PCA versus IR group.

**Fig.S12** Immunofluorescence staining of normal mouse skin. (a) TNF-α staining; (b) VEGF staining; (c) CD86 (red) and F4/80 (green) co-staining; (d) CD206 (red) and F4/80 (green) co-staining. Nuclei were counterstained with DAPI (blue). Scale bar = 100 μm.

Fig.S13 Quantitative analysis of immunofluorescence staining in skin tissues. (a) TNF‑α and (b) VEGF fluorescence intensity; (c) CD86 and CD206 fluorescence intensity normalized to F4/80. Data are mean ± SD (n = 3). One‑way ANOVA with Tukey's test. * p < 0.05, ** p < 0.01, *** p < 0.001.

**Fig.S14** CCK‑8 assay of macrophages 24 h after exposure to different radiation doses (2, 4, 6, and 8 Gy). Data are presented as mean ± SD (n = 3). Statistical significance was determined using one‑way ANOVA followed by Tukey's post‑hoc test. *p < 0.05 vs. the indicated groups.

**Fig.S15** Representative images of macrophage uptake after 4 Gy ionizing radiation for 24 h, followed by co‑incubation with fluorescent microspheres for 1 h, and statistical analysis of uptake rate. Data are presented as mean ± SD (n = 3). Statistical significance was determined using one‑way ANOVA followed by Tukey's post‑hoc test. *p < 0.05, ***p < 0.001.

**Fig.S16** Statistical analysis of ROS flow cytometry in PMs after PCA treatment. Data are presented as mean ± SD (n = 3). Statistical significance was determined using one‑way ANOVA followed by Tukey's post‑hoc test. ****p < 0.0001.

**Fig.S17** Cell viability of macrophages after co-incubation with increasing concentrations of Rotenone (0.1, 0.5, 1.0, 2.5, and 5.0 μM) for (a) 24 h and (b) 48 h. Data are presented as mean ± SD (n = 4). Statistical significance was determined by one-way ANOVA with Tukey's post-hoc test. *p < 0.05, ***p < 0.001.

**Fig.S18** Rotenone blocks PCA-mediated protection of mitochondrial membrane potential. Macrophages were pretreated with PCA for 24 h, followed by irradiation and immediate treatment with Rotenone (1 μM). (a) Cell viability at 12 h post-irradiation. (b) Cell viability at 24 h post-irradiation. (c) Quantification of the JC-1 red/green fluorescence ratio at 12 h and 24 h post-irradiation. (d) Representative JC-1 immunofluorescence staining images of macrophages at 12 h and 24 h post-irradiation (red: aggregates, green: monomers). Data are presented as mean ± SD (n = 3). *p < 0.05, ***p < 0.001, ****p < 0.0001. Statistical significance was determined by one-way ANOVA with Tukey's post-hoc test. Scale bar = 100 μm.

**Fig.S19** Rotenone reverses PCA-induced macrophage polarization and inflammatory modulation. Macrophages were pretreated with PCA for 24 h, followed by irradiation and immediate treatment with Rotenone (1 μM). All assays were performed at 24 h post-irradiation. (a) Representative immunofluorescence images of M1-type markers (CD86 and iNOS). (b, c) Quantification of the percentage of CD86⁺ and iNOS⁺ cells, respectively. (d) Representative immunofluorescence images of M2-type markers (CD206 and ARG1). (e, f) Quantification of the percentage of CD206⁺ and ARG1⁺ cells, respectively. (g) IL-10 levels in cell culture supernatants measured by ELISA. Data are presented as mean ± SD (n = 4). *p < 0.05, **p < 0.01, ***p < 0.001, ****p < 0.0001. Statistical significance was determined by one-way ANOVA with Tukey's post-hoc test. Scale bar = 50 μm.

**Table S2** Molar percentage of monosaccharide content of PSP and OPSP

**Table S3** Primer sequences used in the qRT-PCR for macrophage related genes analysis.

| **Gene** | **5’-3’** | **Primer sequences** |
| --- | --- | --- |
| Actin | Forward primers | GGCTGTATTCCCCTCCATCG |
|  | Reverse primers | CCAGTTGGTAACAATGCCATGT |
| IL-1β | Forward primers | GCCCATCCTCTGTGACTCAT |
|  | Reverse primers | AGGCCACAGGTATTTTGTCG |
| IL-6 | Forward primers | TTCTTGGGACTGATGCTGGTG |
|  | Reverse primers | CACAACTCTTTTCTCATTTCCACGA |
| TNF-α | Forward primers | CAGGCGGTGCCTATGTCTC |
|  | Reverse primers | CGATCACCCCGAAGTTCAGTAG |
| IL-10 | Forward primers | GCTCTTACTGACTGGCATGAG |
|  | Reverse primers | CGCAGCTCTAGGAGCATGTG |
| TGF-β1 | Forward primers | CACTCCCGTGGCTTCTAGTG |
|  | Reverse primers | CTGGCGAGCCTTAGTTTGGA |
| PGC-1α | Forward primers | ATCTTGGAGCGAGTTGTGGATTGTC |
|  | Reverse primers | TAGGTGAGGGCTTGGCTGAGTG |
